# Genome Sequencing Provides Novel Insights into Mudflat Burrowing Adaptations in Eel Goby *Taenioides* sp. (Teleost: Amblyopinae)

**DOI:** 10.3390/ijms241612892

**Published:** 2023-08-17

**Authors:** Yantao Liu, Tianwei Liu, Yuzhen Wang, Jing Liu, Bingjian Liu, Li Gong, Zhenming Lü, Liqin Liu

**Affiliations:** 1 National Engineering Laboratory of Marine Germplasm Resources Exploration and Utilization, College of Marine Sciences and Technology, Zhejiang Ocean University, Zhoushan 316022, China; 2 National Engineering Research Center for Facilitated Marine Aquaculture, Zhejiang Ocean University, Zhoushan 316022, China

**Keywords:** *Taenioides* sp., genome assembly, comparative genomics, mudflat burrowing adaptations

## Abstract

Amblyopinae is one of the lineage of bony fish that preserves amphibious traits living in tidal mudflat habitats. In contrast to other active amphibious fish, Amblyopinae species adopt a seemly more passive lifestyle by living in deep burrows of mudflat to circumvent the typical negative effects associated with terrestriality. However, little is known about the genetic origin of these mudflat deep-burrowing adaptations in Amblyopinae. Here we sequenced the first genome of Amblyopinae species, *Taenioides* sp., to elucidate their mudflat deep-burrowing adaptations. Our results revealed an assembled genome size of 774.06 Mb with 23 pseudochromosomes anchored, which predicted 22,399 protein-coding genes. Phylogenetic analyses indicated that *Taenioides* sp. diverged from the active amphibious fish of mudskipper approximately 28.3 Ma ago. In addition, 185 and 977 putative gene families were identified to be under expansion, contraction and 172 genes were undergone positive selection in *Taenioides* sp., respectively. Enrichment categories of top candidate genes under significant expansion and selection were mainly associated with hematopoiesis or angiogenesis, DNA repairs and the immune response, possibly suggesting their involvement in the adaptation to the hypoxia and diverse pathogens typically observed in mudflat burrowing environments. Some carbohydrate/lipid metabolism, and insulin signaling genes were also remarkably alterated, illustrating physiological remolding associated with nutrient-limited subterranean environments. Interestingly, several genes related to visual perception (e.g., crystallins) have undergone apparent gene losses, pointing to their role in the small vestigial eyes development in *Taenioides* sp. Our work provide valuable resources for understanding the molecular mechanisms underlying mudflat deep-burrowing adaptations in Amblyopinae, as well as in other tidal burrowing teleosts.

## 1. Introduction

The water-to-land transition during the Paleozoic era was a major step in vertebrate evolution and eventually gave rise to the emergence of terrestrial animals [1,2]. After the bony fish firstly showed their terrestriality and active land-invasion, the ancestors of tetrapods successfully moved onto land [1,3]. Such water-to-land transition was usually accompanied by a series of morphological and physiological innovations in the respiratory, sensory, locomotory, circulatory, and other systems [4] that enabled them actively respond to changed environments associated with the terrestriality [5,6,7]. However, some group of presently-living bony fishes evolved rather later seems to tackle this evolutionary dilemma by adopting a much more passive strategy of land conquest by living in tidal mudflat deep-burrows to circumvent the negative effects typically associated with the water-to-land transition. How these bony fishes have evolved to adapt to such mudflat burrowing habitats still remains to be elucidated. 

One group of the bony fishes that show such seemly passive strategy of land conquest by living in mudflat burrow is Amblyopinae (Gobiiformes: Gobionellidae) [2,8], a portion of small elongate, mud-dwelling fishes of the Indo-West Pacific region that are commonly referred to as “eel gobies” [9]. They are usually found in deep burrows of tidal mudflats, muddy bottoms of estuaries, as well as substrates from the adjacent shallow waters [10]. Some species, including taxa in genus *Odontamblyopus* and *Taenioides*, are also frequently observed in intertidal zones in the exposed burrows filled with hypoxia waters during the low tide. The burrows can usually reach to a maximum depth of ~1 m [11]. To better cope with the challenges of deep burrowing environments, Amblyopinae have evolved many morphological and physiological innovations which include small vestigial eyes covered with skin [10,12] that show their adaptations to turbid waters [9], richly vascularized inner epithelia in the buccal-opercular cavity that enable direct air breathing to cope with the hypoxia in their poorly oxygenated burrows [11,13], and improved starvation resistance that ensures survival during long period of embedment in burrows at the time of low tide or cold seasons [11]. However, the genetic basis of such morphological and physiological adaptations in Amblyopinae has remained largely unknown, to date. 

Genome sequencing and comparative genomics is a powerful tool for exploring the evolutionary history and mechanisms of a non-model taxon with complex adaptive traits, such as Amblyopinae. In the present study, we assembled the first genomes of Amblyopinae species, *Taenioides* sp., widely distributed in coastal waters of China [12]. Combined with phylogenomics and comparative genomics, we tried to elucidate the genetic mechanism underlying the morphological and physiological innovations that facilitate the adaptations to deep-burrowing lifestyle in Amblyopinae. The results will largely expand our knowledge on how the deep-burrowing lifestyle is evolved in Amblyopinae, as well as in other tidal burrowing teleost fishes.

## 2. Results

### 2.1. Assembly and Characterization of Taenioides sp. Genome

**Figure 1:**
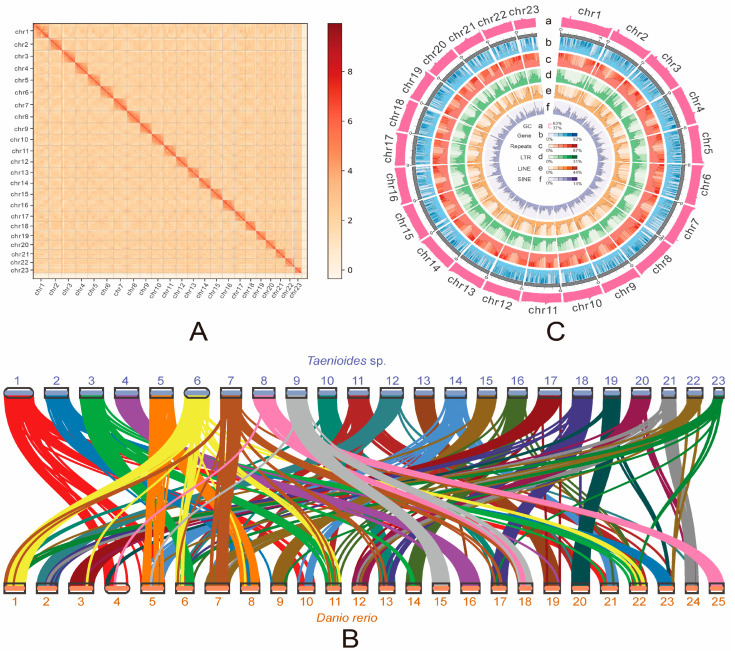
Genome assembly and gene annotation of *Taenioides* sp. (**A**) A heatmap of chromosomal interactions in *Taenioides* sp. The blocks representthe 23 pseudochromosomes. The colour bar illuminates the contact density from blue (low) to red (high). (**B**) Synteny between genomes of *Taenioides* sp. and *D. rerio*. The number in the figure represents the chromosome identity for each species. (**C**) Circos plot of distribution of the genomic elements in *Taenioides* sp. From the outer circle to the inner circle: (a) GC content of the genome; (b) gene distribution; (c) tandem repeats (TRP); (d) long terminal repeats (LTR); (e) long interspersed nuclear elements (LINE); (f) short interspersed nuclear elements (SINE).

Using MGISEQ-T7 short reads sequencing strategies, we generated 75.59 Gb of clean sequencing data, representing 97.79-fold coverage of *Taenioides* sp. genome (Table 1 and Table S1). These MGISEQ-T7 short reads were firstly used to employ the genome survey of *Taenioides* sp. with the 17-mer analysis, which resulted in a total number of 67,447,918,210 k-mers, with a k-mers peak at a depth of 88 (Table S2 and Figure S1). The estimated genome size was ~766 Mb, with heterozygosity of 0.60% and repeat content of 45.32% (Table S2). Then, 37.05Gb clean HiFi CCS sequencing reads with an average length of 18.35 kb (N50: 18.57 Kb; reads number: 2,019,045) were generated, covering 47.93-fold of *Taenioides* sp. genome (Table 1 and Table S3). The genome was pre-assembled with HiFi reads using Hifasm (version 0.16.1), and yielded a reference genome of 774.06 Mb in size with a contig N50 of 19.26Mb (Table 2), which was close to the estimated size of *Taenioides* sp. genome. To acquire the high-quality chromosome-level assembly of the genome, 118.16 Gb Hi-C reads were obtained (Table S4) and further aligned to the pre-assembled reference genome using Juicer (Durand et al., 2016), which resulted in 23 pseudochromosomes with the mounting rate of 97.81% (Figure 1a; Table S5). Chromosome lengths ranged from 18.79 to 43.85 Mb (Table S5). The 23 pseudochromosomes could be distinguished easily based on the heatmap, and the interaction signal around the diagonal was considerably strong, indicating the high quality of this genome assembly (Figure 1a). The completeness of the final assembly of *Taenioides* sp. genome was further assessed using BUSCO, mapping ratio and genome synteny analyses. Results of BUSCO analysis indicated that 96.76% of the complete BUSCO could be found in the assembled genome (Table 3), including 95.82% of the complete and single-copy and 0.93% of the duplicated genes. The mapping ratio analyses indicated that 99.64% of the MGISEQ-T7 short reads and 99.97% of the PacBio HiFi long reads were successfully mapped to the assembled genome (Tables S6 and S7) when we aligned these sequencing reads to the genome. The quality of the chromosome-level assemblies was also demonstrated by the good genome synteny (Table S8; Figure 1b, Figures S2 and S4). Taken together, all these tests proposed that we have obtained a high-quality assembled reference genome of *Taenioides* sp.

A total of 365.87 Mb repetitive sequences were identified in *Taenioides* sp., which accounted for 47.31% of the assembled genome. (Table S9). This repeat content was similar to the value (45.32%) obtained from the k-mer analysis (Table S2). Among these repetitive sequences, DNA elements accounted for the highest proportion (percentage: 12.54%) in the genome among all known transposable elements (e.g., LTRs, DNA elements, LINEs, SINEs), followed by LINEs (percentage: 10.24%), and LTRs (percentage: 6.05%) (Tables S10 and S11). The SINEs accounted for the lowest proportion among the four types of repetitive sequences (percentage: 1.02%) (Tables S10 and S11). In addition, a total of 22,399 protein-coding genes were predicted by the combination of the three annotation strategies based on ab initio, homologues, and RNAseq (Table 4 and Table S12). The characteristics of these predicted genes were compared with the published genome of 4 other teleost species, including *Danio rerio*, *Oryzias latipes*, *Periophthalmus magnuspinnatus* and *Boleophthalmus pectinirostris,* indicating similar value of mRNA length, CDS length, exon length, intron length, and exon number in protein-coding genes among species (Figure S5). Genome characteristics of *Taenioides* sp. were finally summarized in Figure 1c. A total of 21,445 predicted genes (95.74%) were successfully annotated by alignment to the various public annotation databases, including InterPro, NR, Swissprot, TrEMBL, KOG, GO, and KEGG (Table 4). These results provide evidence that we obtained a proper annotation of *Taenioides* sp. genome.

### 2.2. Phylogenetic Origin and Fast Evolution of Taenioides sp.

**Figure 2:**
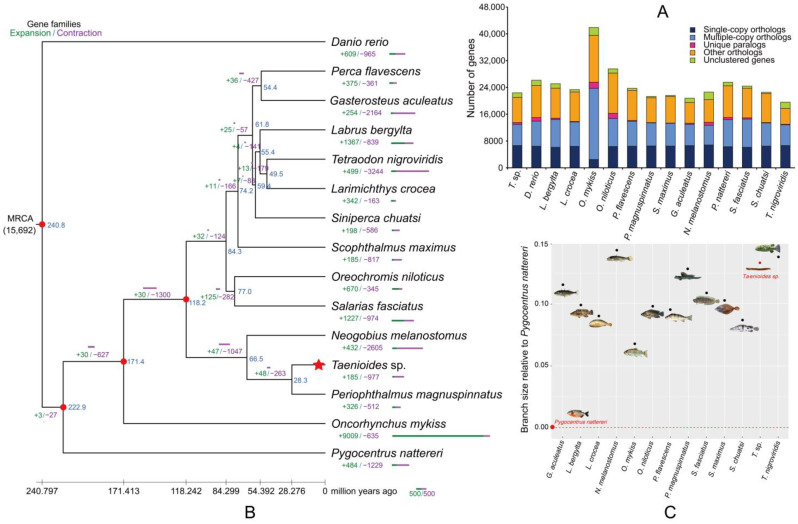
Phylogenetic origin and fast genome evolution of *Taenioides* sp. (**A**) Distribution of different types of orthologues in *Taenioides* sp. and other 14 teleost species. (**B**) Phylogenetic relationship and divergence time among *Taenioides* sp. and other 14 outgroup species. The position of *Taenioides* sp. in the phylogenetic tree was marked with a red star. The blue number in each node represents the divergence time among species and the red circle indicates the fossil record used for calibration in the node. The green and purple numbers in each node, represent the expanded and contracted gene families in this node, respectively. (**C**) Relative evolutionary rates of *Taenioides* sp. and other 14 outgroup species. Zebrafish was used as the outgroup and *Pygocentrus nattereri* as the reference species. The ovals represent different fish groups that showed contrasted relative evolutionary rates with both *Taenioides* sp. and the reference species *Pygocentrus nattereri* marked red.

A total of 1792 single-copy orthologous genes were identified to be shared by *Taenioides* sp. and other 14 outgroup species (*Danio rerio*, *Labrus bergylta*, *Larimichthys crocea*, *Oncorhynchus mykiss*, *Oreochromis niloticus*, *Perca flavescens*, *Periophthalmus magnuspinnatus*, *Scophthalmus maximus*, *Gasterosteus aculeatus*, *Neogobius melanostomus*, *Pygocentrus nattereri*, *Salarias fasciatus*, *Siniperca chuatsi* and *Tetraodon nigroviridis*) (Figure 2A) with the published genome. By combining our *Taenioides* sp. genome with those of 14 outgroup species, we constructed the ML phylogenetic tree among these species using concatenated sequences of coding sequence (CDS) derived from these 1792 single-copy orthologous genes. Our results showed that *Taenioides* sp. formed one clade with the two Gobiiformes species, with it being more closely related to *Periophthalmus magnuspinnatus* (Figure 2B and Figure S6). The observation that *Taenioides* sp. was clustered with the active amphibious fish of *P. magnuspinnatus* rather than other teleost species, suggested that these two terrestrial lineage of the fish may have a common evolutionary origin. This assumption was further supported by results from multiple previous phylogenetic studies, showing their affinity among the two lineages [2,14]. Based on these evidences, Steppan [8] even suggested that these two terrestrial lineage of Amblyopinae and Oxudercinae should be merged into one subfamily under family Gobionellidae of Gobiiformes. 

With fossil calibrations, we estimated the divergence time between *Taenioides* sp. and *P. magnuspinnatus* to be approximately 28.3 million years ago (Ma), in the early Oligocene (Figure 2B). The divergence time was generally consistent with our previously estimated divergence time between subfamily Amblyopinae and Oxudercinae (34.5 Ma) based on mitogenome analyses [2]. The early Oligocene (~34–27  Ma) is widely known as the onset of a global cooling phase, and a worldwide drop in sea level, due to a dramatic atmosphere CO2 decline [15,16]. The global cooling and sharp drop in sea levels have been implicated in the rapid diversification of terrestrial gobies of mudskipper, for low sea level could have offered more mudflats to grow diatoms for the propagation of certain mudskipper species [6]. Whether such a scenario has also contributed to the divergence of *Taenioides* sp. and *P. magnuspinnatus* still awaits further verification. However, this may predict selection pressure and faster evolutionary rate in *Taenioides* sp., compared to other teleost fish, owning to its radical habitat transition from open water column to mudflat deep-burrowing habitats. Our calculations on the relative evolutionary rates of *Taenioides* sp. and other 14 outgroup species based on single-copy orthologous genes seemed to support this hypothesis, because the relative evolutionary rate of *Taenioides* sp. was much higher than that of most outgroup speceis, except for *T. nigroviridis* and *N. melanostomus* (Figure 2C). The higher relative evolutionary rates in *Taenioides* sp. indicated the possible selection pressure it may experience, though additional factors like rapid drift and limited population size can not yet be precluded [17].

### 2.3. Genetic Alterations in Taenioides sp. Possibly Correlated wth Deep-Burrowing Adaptations

**Figure 3:**
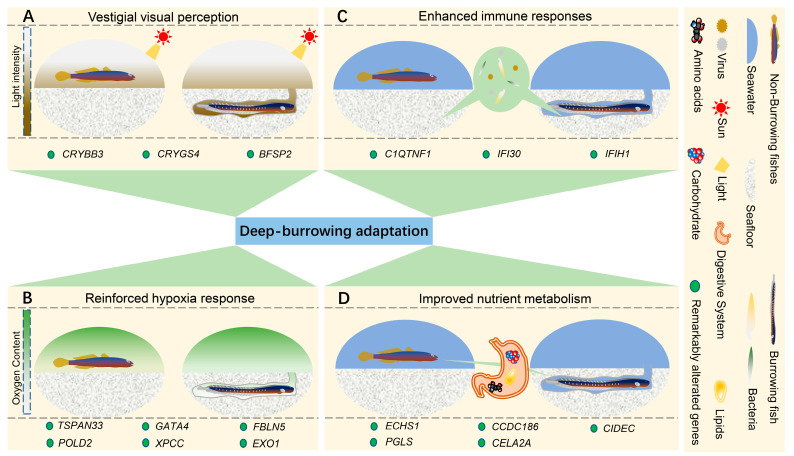
The positively selected genes that may confer deep-burrowing adaptation in *Taenioides* sp. (**A**) shows a scenario of how genes associated with visual perception got lost in *Taenioides* sp. due to their adaptation to turbid waters typically found in the burrowing environment. (**B**) shows how genes associated with hypoxia resistance are under positive selection in *Taenioides* sp. owning to their adaptation to hypoxia in their poorly oxygenated burrows. (**C**) illustrates how genes associated with immune response got positively selected in *Taenioides* sp. owning to their adaptation to diverse pathogens typically observed in deep-burrowing enviroments. Green enlarged diagrams on the panel represent bacteria and viruses. (**D**) elucidates how genes associated with nutrient metabolism are currently under positive selection in *Taenioides* sp. owning to their adaptation to prolonged starvation in nutrient-limited burrowing habitats. Yellow enlarged diagrams on the panel represent nutrient metabolism in the digestive organ.

**Figure 4:**
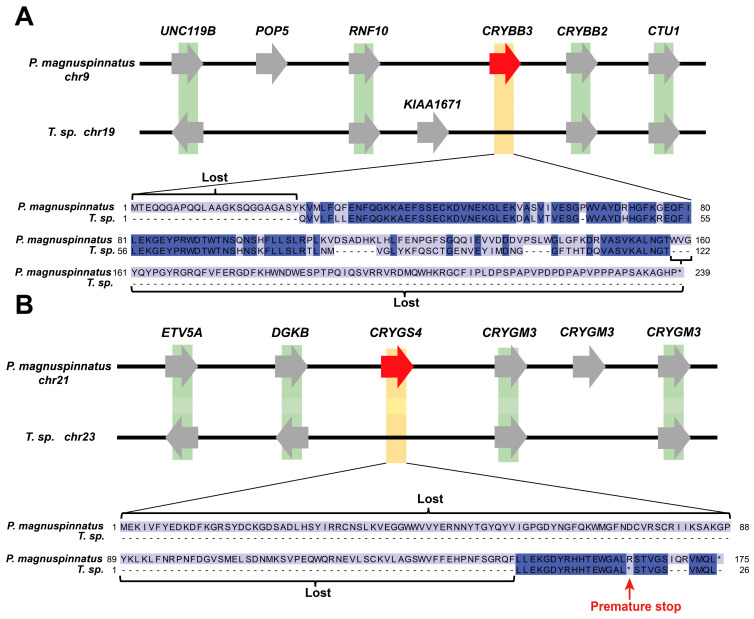
The gene losses (shown in yellow) in *Taenioides* sp. compared with the their closely related species *P. magnuspinnatus*. Both the synteny blocks (Shown in green), lost locus (light blue) and the recognizable conserved residues (blue) in the presumed lost genes were shown. (**A**,**B**) showed the synteny blocks and mutations in the presumed lost genes of *CRYBB3*, and *CRYGS4*.

The higher relative evolutionary rates in *Taenioides* sp. may also predict remarkable alterations in its genome, which had facilitated its radical habitat transition and hence mudflat deep-burrowing adaptations. To test this assumption, a comparative genomic analysis was performed among *Taenioides* sp. and other 14 outgroup species. We first analyzed the expanded and contracted gene families in *Taenioides* sp. genome using CAFE to identify the gene families that changed remarkably in gene number during the evolution process. The results revealed 185 expanded and 977 contracted gene families (*p* < 0.05) in *Taenioides* sp. (Figure 2B). The top expanded gene families were significantly enriched in 43 GO terms and 97 KEGG pathways, which mainly included primary immunodeficiency (ko05340, *p*-value = 9.66 × 10^−5^), B cell receptor signaling pathway (ko04662, *p*-value = 3.53 × 10^−11^), Hematopoietic cell lineage (ko04640, *p*-value = 4.10 × 10^−12^), complement and coagulation cascades (ko04610, *p*-value = 1.05 × 10^−5^), Type I diabetes mellitus (ko04940, *p*-value = 1.17 × 10^−4^), fatty acid degradation (ko00071, *p*-value = 3.88 × 10^−3^) that were associated with immune response, hematopoiesis or angiogenesis, and nutrient metabolism (Tables S13 and S14). Conversely, the top contracted gene families were significantly enriched in neurotransmitter transport (GO:0006836, *p*-value = 1.17 × 10^−4^), neurotransmitter:sodium symporter activity (GO:0005328, *p*-value = 7.27 × 10^−5^), NOD-like receptor signaling pathway (ko04621, *p*-value = 2.74 × 10^−13^) that were maily related to neurotransmission and immune response (Tables S15 and S16). We further identified 172 genes undergone positive selection (PSGs) in *Taenioides* sp. (Table S17). The enrichment categories of the top PSGs were associated with hematopoiesis or angiogenesis (*TSPAN33*, *GATA4*, and *FBLN5*) [18,19,20], DNA repairs (*POLD2*, *XPCC*, and *EXO1*) [21,22,23], immune response (*C1QTNF1*, *IFI30* and *IFIH1*) [24,25,26], and carbohydrate/lipid metabolism (*PGLS*, *ECHS1*, *CIDEC, CELA2A,* and *CCDC186*) [27,28,29,30,31] (Figure 3, Tables S18 and S19). The evidence of positive selection in genes associated with hematopoiesis or angiogenesis, DNA repairs, immune response, and carbohydrate/lipid metabolism may indicate a role of remolding of circulatory, immune, and metabolic systems of *Taenioides* sp. in adaptation to the hypoxia, diverse pathogens and limited nutrition typically observed in mudflat deep-burrowing environments. Using the *P. magnuspinnatus* genome as a reference, we also detected three orthologous genes (*CRYBB3*, *CRYGS4*, and *BFSP2*) [32,33,34] associated with visual perception undergone gene losses in *Taenioides* sp. Among them, two of them (*CRYBB3*, *CRYGS4*) have recognizable conserved residues based on futher manual inspection (Figure 3 and Figure 4). The observed lost genes associated with the visual perception appears to underlie the evolution of vestigial eyes in *Taenioides* sp.

## 3. Discussion

### 3.1. Possible Genetic Origin of Visual Modification Adapted to Burrowing Habitats in Taenioides sp.

The observed lost genes associated with eye development and visual perception may point to their roles in the genetic origin of small vestigial eyes in *Taenioides* sp. The phenotype of small vestigial eyes is a typical characteristics of Amblyopinae species, which is usually recognized as a regressive feature adapted to turbid waters in burrowing environments [9]. However the genetic mechanism underlying it has not yet been elucidated. Our comparative genomic analyses using the *P. magnuspinnatus* genome as a reference, revealed that three genes associated with visual perception have undergone gene losses (but the conserved residue of the lost gene are still recognizable in only two of them) (Figure 4) in *Taenioides* sp., including Beta-crystallin B3 gene *CRYBB3*, Phakinin gene *BFSP2*, and Gamma-crystallin S4 gene *CRYGS4*. All three genes encode either the dominant structural components of the vertebrate eye lens [33,34], or intermediate filaments in lens that are indispensable for visual perception and eye development [32]. Among them, Gamma-crystallin S4 is restricted to the optic lens or retina of the aquatic animals including fishes [34] and functions as important molecules involved in the visual perception pathway. Phakinin is commonly found exclusively in the lens fibre cells, comprising a unique cytoskeletal structure called the beaded filament, and being essential for lens function and specifically contributing to the optical properties of the lens [32]. Genetic disruption or depletion of *BFSP2* will result in severe visual impairment, such as myopia [32], congenital cataract [35], loss of lens function [36] in both human and mice. Beta-crystallin B3 is one important form of β-crystallins that are necessary for lens transparency and eye development of vertebrates [33,37]. Mutations or abnormal expression in *CRYBB3* would lead to severe developmental disorders of eye development, such as poor vision [38], cataract [35], corneal degeneration [39], or some form of microphthalmia (syndrome with unusual smaller eyes) [39,40] in Humans. Such gene losses in genome may change the visual perception or eye development in animals, and may be implicated in the evolution of small vestigial eyes in *Taenioides* sp, though the causative effect of these presumed gene losses still awaits further investigation. 

### 3.2. Possible Genetic Origin of Improved Hypoxia Resistance Adapted to Burrowing Habitats in Taenioides sp.

High hypoxia resistance is another striking feature of *Taenioides* sp. To cope with the frequent hypoxia conditions in their poorly oxygenated burrows, *Taenioides* sp. has evolved richly vascularized inner epithelia in the buccal-opercular cavity that enables thier direct air breathing when oxygen is limited, yet its genetic basis remains largely unknown. Our comparative genomic analyses revealed that the gene families associated with hematopoiesis or angiogenesis were largely expanded in *Taenioides* sp. (Tables S13 and S14). Furthermore, three genes related to hematopoiesis or angiogenesis were also undergone positive selection in *Taenioides* sp. These three genes include *TSPAN33* (PSGs, *p*-value = 1.70 × 10^−4^), *GATA4* (PSGs, *p*-value = 8.70 × 10^−4^) and *FBLN5* (PSGs, *p*-value = 3.70 × 10^−3^) (Figure 3, Table S17), which encode proteins that are essential for normal erythropoiesis [18], and vascular development [19,20]. Mutation or expression disruptions of these three genes would lead to improved erythropoiesis, angiogenesis and increased micorvascular density in systemic and cutaneous circulatory systems in vertebrates [18,19,20]. Such remarkable changes in these hematopoiesis or angiogenesis related genes may suggest their roles in the evolution of richly vascularized inner epithelia and hence their high hypoxia resistance of *Taenioides* sp. In addition, the substantial alterations in genes associated with DNA repairs (*POLD2*, *XPCC*, and *EXO1*) (Table S17) may further reinforce the hypoxia resistance in *Taenioides* sp., due to their functionally closely related between the two system [41]. Hypoxia have long been recognized as one of critical environmental factors inducing aerobic metabolism and hence burst of reactive oxygen species (ROS), which are harmful to living cells, leading to DNA damage, senescence, or cell death [41]. Such ROS-mediated DNA damage can be repaired by celluar DNA repairing system consisting of proteins involved in variety of repairing parthways [42]. The observed positive selection in genes associated with DNA repair system in *Taenioides* sp. (Figure 3, Table S17) may reflect the necessity of improved DNA repair efficiency that circumvent the negative effects typically associated with the hypoxia, though other merit of these mutations, such as maintaining genomic stability responding to harsh temperature gradients in tidal zone [6], could not yet be excluded. 

### 3.3. Genetic Changes Possibly Confer Enhanced Immune Response Adapted to Burrowing Habitats in Taenioides sp.

Coinciding with the mudflat deep-burrowing lifestyle, *Taenioides* sp. seems to have also evolved an enhanced immune system to cope with the diverse pathogens typically observed in mudflat substrate [43], since our comparative genomic analyses revealed that multiple gene families associate with immune response were largely expanded in *Taenioides* sp. Actually immune genes were the most expanded and overpresented genes in the genome of *Taenioides* sp. compared with their counterparts (Tables S13 and S14). Furthermore, three genes were also observed under positive selection in *Taenioides* sp. (Figure 3, Table S17), which includes *C1QTNF1* (PSGs, *p*-value = 2.04 × 10^−3^), *IFI30* (PSGs, *p*-value = 3.09 × 10^−4^), and *IFIH1* (PSGs, *p*-value = 7.39 × 10^−8^), indispensable for innate immune defence against pathogens [24,25,26]. Mutations or expression disruption of these genes has been confirmed to prominently change the immune resistance against pathogens in teleosts [24,26]. Some mutations of these genes have even implicated in susceptibility of certain autoimmune disease in multiple vertebrate species, including humans [44,45]. Therefore, the remarkable alterations in these three genes may provide special immune defence against diverse pathogens typically observed in deep-burrowing enviroments. However, this hypothesis has to be verified by comparing with the genomes of more non-burrowing gobies and determining whether the alterations of these genes have occurred specifically in Amblyopinae species. 

### 3.4. Genetic Changes Possibly Confer Altered Nutri-Physiology Adapted to Burrowing Habitats in Taenioides sp.

Interestingly, our comparative genomic analyses also revealed five genes associated with nutrient (carbohydrate/lipid) metabolism that has undergone remarkable changes in *Taenioides* sp., including *ECHS1* (PSGs, *p*-value = 2.18 × 10^−10^), *CIDEC* (PSGs, *p*-value = 2.95 × 10^−10^), *PGLS* (PSGs, *p*-value = 1.78 × 10^−3^), *CELA2A* (PSGs, *p*-value = 1.92 × 10^−4^), and *CCDC186* (PSGs, *p*-value = 1.03 × 10^−3^) (Figure 3, Table S17). Among them, *ECHS1* and *CIDEC* encode enzymes or metabolic signals essential for lipogenesis and fat accumulation in vertebrates [28,31]. Mutations or abnormal expressions of *ECHS1* and *CIDEC* can result in increased adiposity and fat deposition in both human [46] and mice [28,31]. Similarly, *PGLS*, *CELA2A*, and *CCDC186* encode molecules that play important roles in carbohydrate degradation [27] and cellular glucose intake [29,30]. Mutations of *PGLS*, *CELA2A*, and *CCDC186* would lead to deficiency in glucose metabolism and energy homeostasis in various organism [27,29], including human [30]. A more close inspection of these carbohydrate metabolic genes indicated that *CELA2A* and *CCDC186* may actually exert their effects through regulation of a network of insulin signalling pathway [29,30]. Such alterations in insulin signalling have typically been observed in vertebrates living in subterranean enviroments [47], including cavefishes [48,49], and these changes were believed to confer elevated appetite, growth, and starvation resistance in animals living in these nutrient-limited habitats. We suspect that the observed alterations in these nutrient metabolism genes in *Taenioides* sp. are also likely beneficial in the nutrient-limited burrowing habitats and may play an important role in circumventing the typical negative effects of prolonged starvation to ensure their survival during long period of time of embedment in burrows at low tide or cold seasons. 

### 3.5. Future Directions for Pursuing the Burrowing Adaptation Mechanisms in Taenioides sp.

Though, as described above, several pieces of evidences have been revealed possibly underlying the mudflat burrowing adaptations using our comparative genomic analyses, further more detailed studies are needed in order to improve our understanding on this aspect in *Taenioides* sp. in the future. Firstly, future’s studies should involve the functional characterization of the genes highlighted in this study to reveal the causative effect of these presumed altered genes associated with the mudflat burrowing adaptations in *Taenioides* sp. That is critical important because comparative genomic analyses are powerful in capturing genes highlighted in the genetic adaptation, but can provide limited information on how these genes work to finally endow the advantages for adaptations. From these point of view, the genes highlighted in our comparative genomic analyses may lay a blueprint for future functional characterization of the molecular mechanisms underlying the mudflat burrowing adaptation in *Taenioides* sp. Secondly, future’s studies should also include both genetic relaxation and positive selection to real broader genetic mechanisms underlying the mudflat burrowing adaptation in *Taenioides* sp. This is also crucial because several lines of evidences have proved that the adaptation to passive lifestyle in subterranean environments may also involve relaxation of natural selection, in addition to pervasive positive selection [50,51], including in subterranean cave fishes [52]. In deed, Rétaux and Casane [53] reviewed the literature describing developmental biology and molecular evolution studies in order to examine the evolutionary mechanisms underlying adaptation to subterranean environments, especially distinctive for adaptations to darkness. They finally concluded that both genetic relaxation and positive selection occurred together during the evolutionary adaptations to subterranean environments. Therefore, future’s analysis should move towards to capture more genome-wide signatures, involving both neutral (genetic relax and gene losses) and positive process to understand the genetic mechanism underlying the mudflat burrowing adaptation in wider taxa of Amblyopinae species, in addition to *Taenioides* sp. 

## 4. Materials and Methods

### 4.1. DNA and RNA Extraction

A female adult *Taenioides* sp. (~30 g in weight) was collected in June 2021 from the intertidal zone of Chongming Island, Yangze estuary of China. Muscle, eye, gill, and liver tissues were collected and stored in liquid nitrogen until DNA and RNA extraction. Genomic DNA was isolated from muscle tissues using Blood & Cell Culture DNA Mini Kit (QIAGEN, Cat. No. 13343). Total RNA was extracted using TRIzol reagent (Invitrogen, Carlsbad, CA, USA). The quality and concentration of extracted DNA/RNA were assessed using a Pultton DNA/Protein Analyzer (Plextech, New York, NY, USA), and their integrity was further evaluated on 1% agarose gel stained with ethidium bromide. The extracted DNA/RNA were stored at −80 °C until use. All tissue collection and DNA/RNA extraction processes followed all relevant ethical regulations provided by the Institutional Animals Care and Use Committee of Zhejiang Ocean University.

### 4.2. Library Constructions and Sequencing

For genome assembly of *Taenioides* sp., PacBio HiFi sequencing libraries were prepared with the SMRTbell Express Template Prep Kit 2.0 (PacBio 101-843-100) using the PacBio low input protocol (DNA sheared to 15 kb), followed by a final clean-up and concentration step using AMPure PB beads (Pacific Biosciences, Menlo Park, CA, USA). The libraries were then sequenced on two PacBio Sequel II SMRT cells in CCS mode on the PacBio Sequel II system. Short-insert (350–700 bp) paired-end libraries were also constructed to correct and evaluate the assembly from the extracted genomic DNA of *Taenioides* sp. using the MGIEasy FS DNA Library Prep Kit (BGI, Cat. No.1000006988) and sequenced on the MGISEQ-T7 platform. Genome of *Taenioides* sp. was further sequenced on the Hi-C platform to obtain chromosome-level genome assemblies. The Hi-C libraries were constructed using genomic DNA extracted from *Taenioides* sp. after fragmented and purified using magnetic beads. For genome annotation of *Taenioides* sp., the complementary DNA libraries were constructed from RNA extracted from four tissues of the eye, liver, muscle, and gill according to the manufacturer’s instructions (BGI) and sequenced on the MGISEQ-T7 platform.

### 4.3. Genome Assemblies and Chromosome Construction

Firstly, MGISEQ-T7 short reads were used to estimate the genome size, heterozygosity and repeat content based on k-mer analyses. Briefly, quality control of DNBSEQ-T7 short reads was performed and the low-quality reads, duplicated reads and adapter sequences were removed using fastp (version 0.23.2) [54]. Distribution of 17-mer frequency was obtained using clean reads by GCE software (version 1.0.0) [55]. GenomeScope 2.0 [56] was then applied for the estimation of genome size and other features such as rate of heterozygosity and abundance of repetitive elements based on these k-mer data. Secondly, the HiFi CCS sequencing reads were used for the genome assembly of *Taenioides* sp. All low-quality reads and adapter sequences were removed using the PacBio SMRT-Analysis before the assembly. Clean reads were then assembled into contigs using Hifiasm (version 0.16.1) [57] with the option for aggressive duplicate purging enabled (option -l 2). Thirdly, to acquire high-quality, chromosome-level assembly of *Taenioides* sp. genome, Hi-C reads were aligned to the pre-assembled genome using Juicer [58]. 3D de novo assembly (3D-DNA) pipeline [59] with default parameters was subsequently used for the chromosomal-level genome assembly by using the corrected contigs and valid Hi-C reads. Scaffolds were then manually refined with Juicebox (version 1.11.08) [58] and visualized in R ggplot2 package to evaluate the quality of the chromosomal-level genome assembly. Finally, the completeness and accuracy of the genome assembly were assessed using BUSCO, reads mapping ratio and genome synteny analyses. For BUSCO analysis, Benchmarking Universal Single-Copy Orthologs (BUSCO) software (version 5.3.1) [60] was applied to evaluate the quality of genome assemblies, with both the eukaryotic and metazoan databases used. For reads mapping ratio analyses, the sequencing reads from both PacBio HiFi sequencing libraries and MGISEQ-T7 short-insert libraries were aligned to the assembled genomes using the Minimap (version 2.2.22) [61] and BWA (version 0.7.12) [62] software, and the mapped read numbers and the mapping ratios were calculated respectively. For genome synteny analysis, MUMmer software (version 4.0.0) [63] was used to align the assembled genomes to the published reference genomes of *Danio rerio*, *Larimichthys crocea* and *Salarias fasciatus* and the best one-to-one aligned blocks were obtained. The synteny relationships between these paired genomes were displayed in circos plots to evaluate the quality of genome assembly.

### 4.4. Genome Annotations

Firstly, repetitive sequences in the genome were identified using different software combinations as follows. Tandem repeats were annotated using the Tandem Repeat Finder software (version 4.09) [64] with default parameters. Non-interspersed repeats were searched using the RepeatMasker software (version 4.0.9) [65] with default parameters except for “-noint”. Transposable elements (TEs) were annotated on both DNA and protein levels. On the DNA level, RepeatModeler software (version 1.0.11) [66] was used to build de novo repeat library and RepeatMasker (version4.0.9) [65] was then run against the de novo library and repbase (RepBase v.16.02) separately to identify homologous repeats. On the protein level, the RepeatProteinMask (RM-BLASTX) [65] was used to search TEs in its protein database. 

Secondly, protein-coding genes were annotated using a combined strategy of ab initio, transcript evidence and protein homology-based methods. For ab initio prediction, Augustus (version 3.3.2) [67] and GENSCAN (version1.0) [68] were used. For transcript-based annotation, cleaned RNA-seq reads were assembled into transcripts using Bridger (r2014-12-01) [69] and aligned against the assembled genomes using BLAT (version 34, identity > 90%, coverage > 90%) [70], and PASA (version 2.1.0) [71] was then used to link spliced alignments. For homology-based annotation, the *Taenioides* sp. genome was aligned against the published *Danio rerio*, *Oryzias latipes*, *Periophthalmus magnuspinnatus* and *Boleophthalmus pectinirostris* genomes using TBLASTN (version 2.11.0) [72] with an e-value cutoff of 1 × 10^−2^. GeneWise (version 2.4.1) [73] was then employed for structural inspection of these alignments to identify the longest coding regions and/or highest score in each gene locus to support the presence of a homologous gene. Finally, the predictions obtained with these three methods were combined using Maker2 (version 2.31.10) [74]. The predicted genes were functionally annotated using the public protein databases: NR, TrEMBL, SwissProt, KEGG, InterPro, KOG, and GO. InterproScan (version 5.55) [75] was used for protein function prediction based on the conserved protein domains (Pfam, version 27.0; prints, version 42.0; prosite, version 20.97; ProDom, version 2006.1; smart, version 6.2). The Kyoto Encyclopedia of Genes and Genomes (KEGG) [76], NR, SwissProt (version 2011.6) [77] and TrEMBL (version 2011.6) [77] databases were also searched for homology-based function assignments using BLAST software (version 2.2.26) [72] with e-value of 1 × 10^−5^.

### 4.5. Phylogenetic Tree Constructions and Divergence Time Evaluation

Orthologs were identified in the assembled genome of *Taenioides* sp., along with the teleost species with published genome sequences (*Danio rerio*, *Labrus bergylta*, *Larimichthys crocea*, *Oncorhynchus mykiss*, *Oreochromis niloticus*, *Perca flavescens*, *Periophthalmus magnuspinnatus*, *Scophthalmus maximus*, *Gasterosteus aculeatus*, *Neogobius melanostomus*, *Pygocentrus nattereri*, *Salarias fasciatus*, *Siniperca chuatsi* and *Tetraodon nigroviridis*) using the OrthoMCL pipeline (version 2.0.9) [78]. Only the longest transcript was chosen for each gene locus with alternative splicing variants. All the single-copy homologous genes shared by all 15 species were further aligned using MUSCLE (version 3.8.31) [79] and concatenated into supergenes for phylogenetic relationship analyses. Maximum likelihood-based phylogenetic analysis was carried out using RAxML (version 8.2.12) [80]. Divergence time among species was then estimated via Bayesian relaxed molecular clock approach using MCMCtree program in the PAML package (version4.8) [81]. Fossil records of the divergence times of *Danio rerio*–*Salarias fasciatus* (180.0–264.0 million years ago (Ma), *Periophthalmus magnuspinnatus*–*Salarias fasciatus* (50.0–121.6 Ma) and *Oncorhynchus mykiss*–*Scophthalmus maximus* (165.0–264.0 Ma) from the TIMETREE website (http://www.timetree.org (accessed on 18 March 2023)) [82] were used for calibrating our calculated divergence time.

### 4.6. Estimation of Relative Evolutionary Rates

The relative evolutionary rates of *Taenioides* sp. compared with other 14 outgroup species were calculated using two-cluster analysis and Tajima’s relative rate test in the LINTRE [83] and MEGA (version 7.0.14) [84] softwares, respectively. For two-cluster analysis, LINTRE program [83] was used to test molecular evolution of multiple sequences in the phylogenetic context and the Z-statistics and tpcv module in LINTRE were used to estimate a faster or slower evolutionary rate in a particular taxon. For Tajima’s relative rate test, MEGA (version 7.0.14) was performed to calculate the speices-specific substitutions, and a higher number of species-specific substitutions indicate a much faster evolutionary rate using the chi-squared test. All the single-copy homologous genes shared by all 15 species were used in these two analyses with *Pygocentrus nattereri* used as referrence.

### 4.7. Estimation of Gene Family Expansion and Contraction

Gene family expansion and contraction in *Taenioides* sp. were determined using the CAFE software (version 4.2) [85]. The phylogenetic tree and divergence time analyzed in the previous steps was used in CAFE to infer changes in gene family sizes using a probabilistic model. *p*-value < 0.05 was used to indicate significantly changed gene families. GO and KEGG enrichment analyses of these expanded and contracted gene families were performed using Enrich GO and Rscript. Significantly over-represented GO terms and KEGG pathways were identified with *p*-values ≤ 0.05.

### 4.8. Detection of Positive Selection

All one-to-one single-copy orthologous genes identified in *Taenioides* sp. and other 14 outgroup species were analysed to detect signals of positive selection. The CODEML program in software PAML package (version 4.8) [81] was used to estimate the dN/dS ratio (the ratio of nonsynonymous substitutions to synonymous substitutions) using branch model. The species-specific positively selected genes (PSGs) were identified with *Taenioides* sp. as foreground and other 14 outgroup species as background. χ^2^ tests were used to determine the significantly positively selected gene with the threshold *p*-value < 0.05. GO and KEGG enrichment analyses of these PSGs in *Taenioides* sp. were also performed using Enrich GO and Rscript, with significantly over-represented GO terms and KEGG pathways identified with *p*-values ≤ 0.05. 

### 4.9. Identification of Gene Losses

Basically, gene losses in *Taenioides* sp. are defined as gene present in the genome of closely related species of *P. magnuspinnatus* but unable to find homologs in *Taenioides* sp. genome. Specially, in this study, we focus on about 280 genes that are associated with visual perception in human and fishes (GO:0007601) [50] to reveal the genetic mechanism underlying the unique vestigial eyes development in *Taenioides* sp. The identification of gene losses was conducted according to the methods decribed by Yu [86]. Briefly, syntenic orthologous gene pairs and syntenic blocks were identified using the QUOTA-ALIGN package [87]. Gene located in *Taenioides* sp.—*P. magnuspinnatus* synteny block and present in syntenic block of *P. magnuspinnatus* genome but unable to find homologs within five syntenic gene pairs of corresponding *Taenioides* sp. syntenic blocks were considered as “potential lost gene”. Several strict filtering steps were executed to remove the possible false positive gene losses due to genome assembly and/or annotation errors, which include: (1) These potential lost genes were first checked in unanchored scaffolds or contigs to exclude false gene losses due to the misassembly. (2) The potential lost genes was further mapped in *P. magnuspinnatus* to the corresponding syntenic DNA sequences in *Taenioides* sp. to remove false gene losses due to the potentially missannotated gene. The possible miss-annotated gene were identified using a homolog-based strategy in *Taenioides* sp. using GeMoMa [88]. The potential lost gene was considered a false positive if the newly predicted gene was homologous to the reference gene at an identity ≥ 80% with coverage ≥ 80% and the gene locus was within five adjacent syntenic gene pairs. (3) These potential lost genes were further validated using raw Illumina short reads generated from the same accession. To validate the lost gene by short reads, BWA (version 0.7.12) [62] was used to map the clean Illumina reads from *Taenioides* sp. to genome of *P. magnuspinnatus.* The depth was calculated using command “bedtools coverage -counts” for each lost gene. Only genes with depth < 1× and coverage of gene body < 5% were recognized as true lost genes. (4) Furthermore, both the lost locus and the recognizable conserved residues in the presumed lost genes were further manually inspected to validate the potential lost genes. 

## 5. Conclusions

Our study, for the first time, assessed the genomic characteristics of *Taenioides* sp, a representative Amblyopinae species which adopts a passive strategy of land conquest by living in mudflat burrows. By comparing with the genomic data of the non-Amblyopine teleost species, we revealed the unique genomic mechanism for their deep-burrowing adaptations. In order to better adapt to the hypoxia, diversified pathogens, and poor nutrients typically encountered in mudflat deep-burrowing habitats, *Taenioides* sp. genome has undergone substantial alterations in genes associated with hypoxia resistance, immune response, nutrient metabolism and insulin signalling. In addition, some visual perception (e.g., crystallins) genes have undergone obvious gene losses, pointing to their role in the vestigial eyes development in *Taenioides* sp. Our results have not only shed insights on the genomic mechanisms underlying the unique habitat adaptations in mudflat deep-burrowing teleosts, such as Amblyopinae, but also provided an alternative evolutionary scenario and mechanism on how teleost fishes could possibly move onto land by adopting a passive burrow-inhabiting strategy during the evolution. More studies are still needed in order to improve our understanding on broader genomic mechanisms of mudflat deep-burrowing in Amblyopinae in the future. 

## Figures and Tables

**Table 1:** Statistics of the cleaned genomic sequencing data for *Taenioides* sp.

Nucleotide Type	Sequencing Strategy	Library Size (bp)	Clean Data (Gb)	Sequencing Depth (x)
Genome	PacBioHiFi	15,000	37.05	47.93
Genome	MGISEQ-T7	300	75.59	97.79
Genome	Hi-C	300	118.16	152.86
RNA	MGISEQ-T7	300	6.61	—

**Table 2:** Statistics of the assembled genome of *Taenioides* sp. based onthe HiFi and Hi-C data.

Term	HiFiasm Contigs		Hi-C Scaffolds	
	Size (bp)	Number	Size (bp)	Number
N90	2,215,384	52	27,022,322	21
N80	6,471,807	34	29,825,879	18
N70	9,977,670	25	31,731,700	16
N60	16,903,772	19	33,492,621	13
N50	19,260,084	15	34,184,133	11
Max length (bp)	40,497,657		43,849,507	
Total length (bp)	774,058,921		773,284,894	
Total number (>100 bp)	287		106	
Total number (>10 kb)	287		106	

**Table 3:** Assessment of genomeassembly quality for *Taenioides* sp. based on BUSCO analysis.

	Gene Number	Percentage
Complete Buscos	3522	96.76
Complete and single-copy BUSCOs	3488	95.82
Complete and duplicated BUSCOs	34	0.93
Fragment BUSCOs	21	0.58
Missing BUSCOs	97	2.66
Total BUSCOs groups searched	3640	100.00

**Table 4:** Statistics of functional annotate protein-coding genes in *Taenioides* sp.

Database	Annotated Gene Number	Percentage (%)
Go	15,263	68.14
INTERPRO	19,975	89.18
KEGG	21,053	93.99
SWISSPROT	19,194	85.69
TREMBL	21,270	94.96
All annotated genes	21,445	95.74
All predicted genes	22,399	100.00

## Data Availability

All raw sequencing data (PacBio HiFi, MGISEQ-T7, Hi-C and RNA-seq data) for *Taenioides* sp. genome was deposited at the NCBI in the sequence read archive (SRA) under accession number (BioProject Number: PRJNA955955). The assembled genome are openly available in figshare at: https://doi.org/10.6084/m9.figshare.22799645 (accessed on 11 May 2023 by Liu and Lü). The results from 1:1 ortholog analysis and CAFE analysis are also provided in figshare at: https://doi.org/10.6084/m9.figshare.23828814.v1 (accessed on 3 August 2023 by Liu).

## Supplementary Materials

The following supporting information can be downloaded at: https://www.mdpi.com/article/10.3390/ijms241612892/s1.
