# Supplementary material for: Genome Sequencing Provides Novel Insights into Mudflat Burrowing Adaptations in Eel Goby Taenioides sp. (Teleost: Amblyopinae)
Source: Int J Mol Sci. 2023 Aug 17;24(16):12892. doi: 10.3390/ijms241612892 (PMC10454203; doi:10.3390/ijms241612892)
Supplement: Supplementary file 1 [file ijms-24-12892-s001.zip › ijms-2550818-Supplementary Figures.pdf]

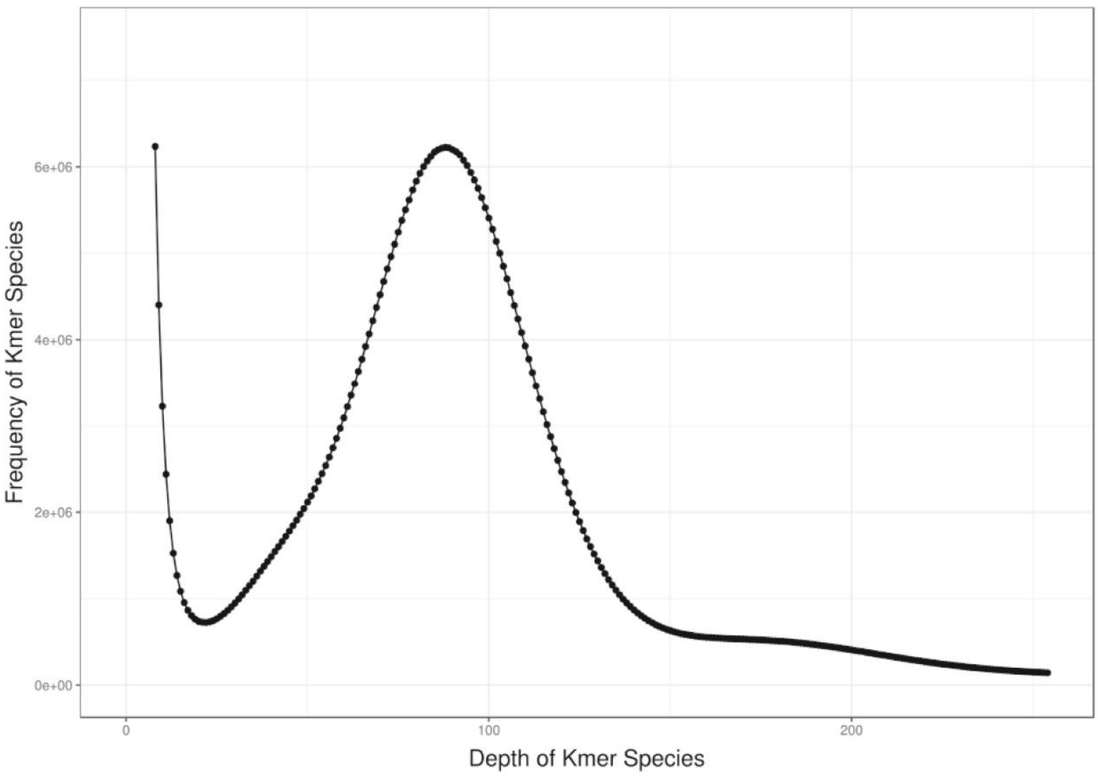

Figure S1 The genome survey for *Taenioides* sp based on 17-mer analysis.

Note: The k-mers peak at a depth of 88.

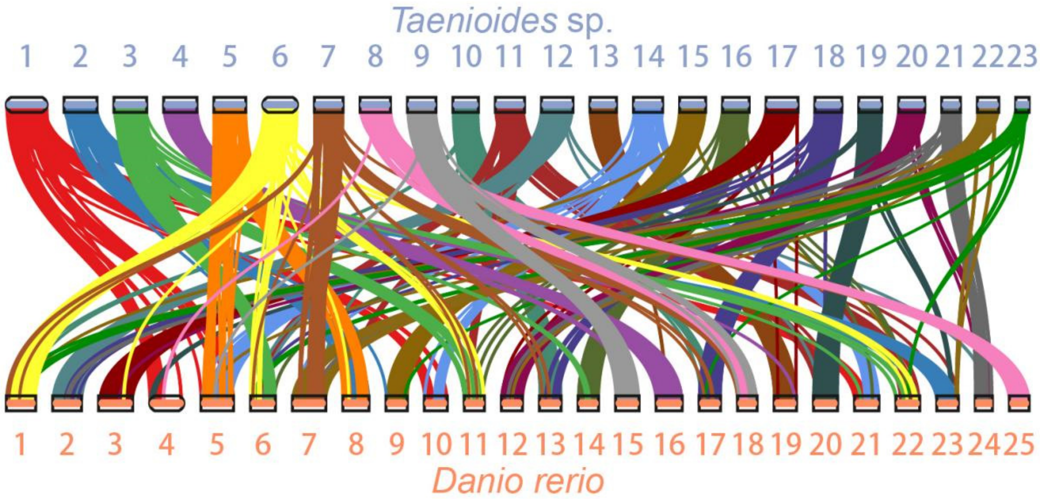

Figure S2 Synteny between genomes of *Taenioides* sp and *Danio rerio*

Note: The number in the figure represents the chromosome identity for both species.

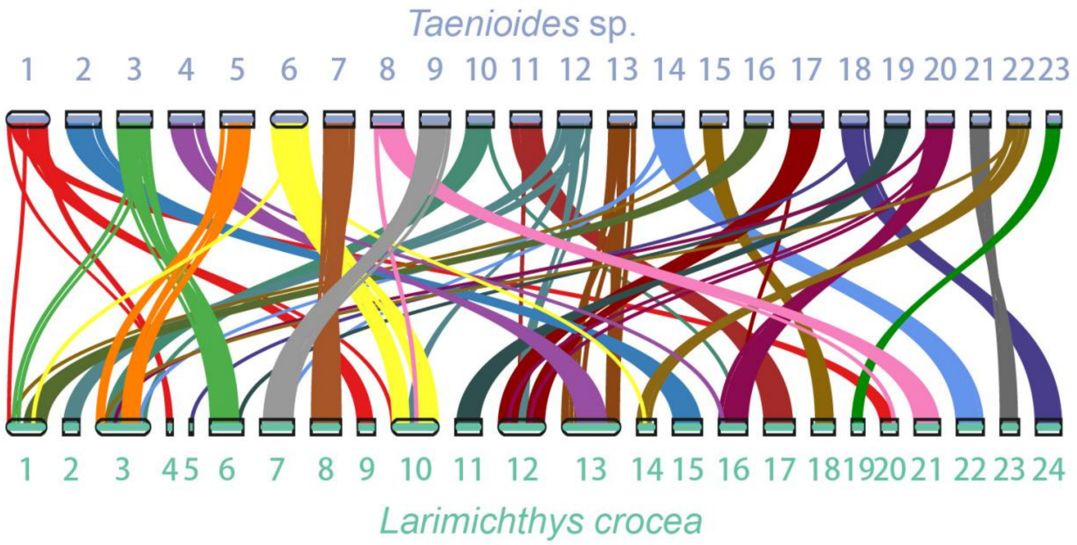

Figure S3 Synteny between genomes of *Taenioides* sp and *Larimichthys crocea*

Note: The number in the figure represents the chromosome identity for both species.

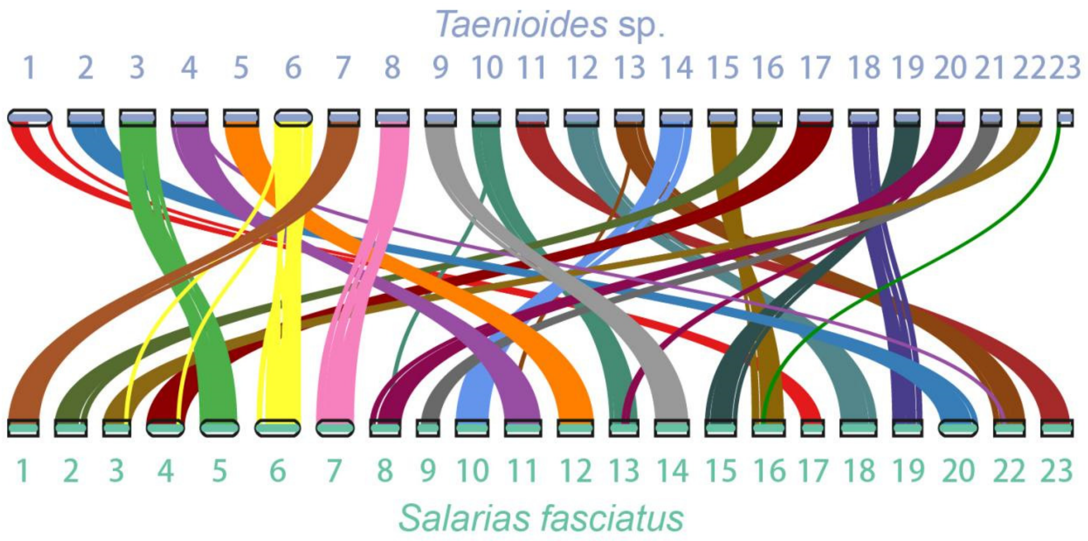

Figure S4 Synteny between genomes of *Taenioides* sp and *Salarias fasciatus*

Note: The number in the figure represents the chromosome identity for both species.

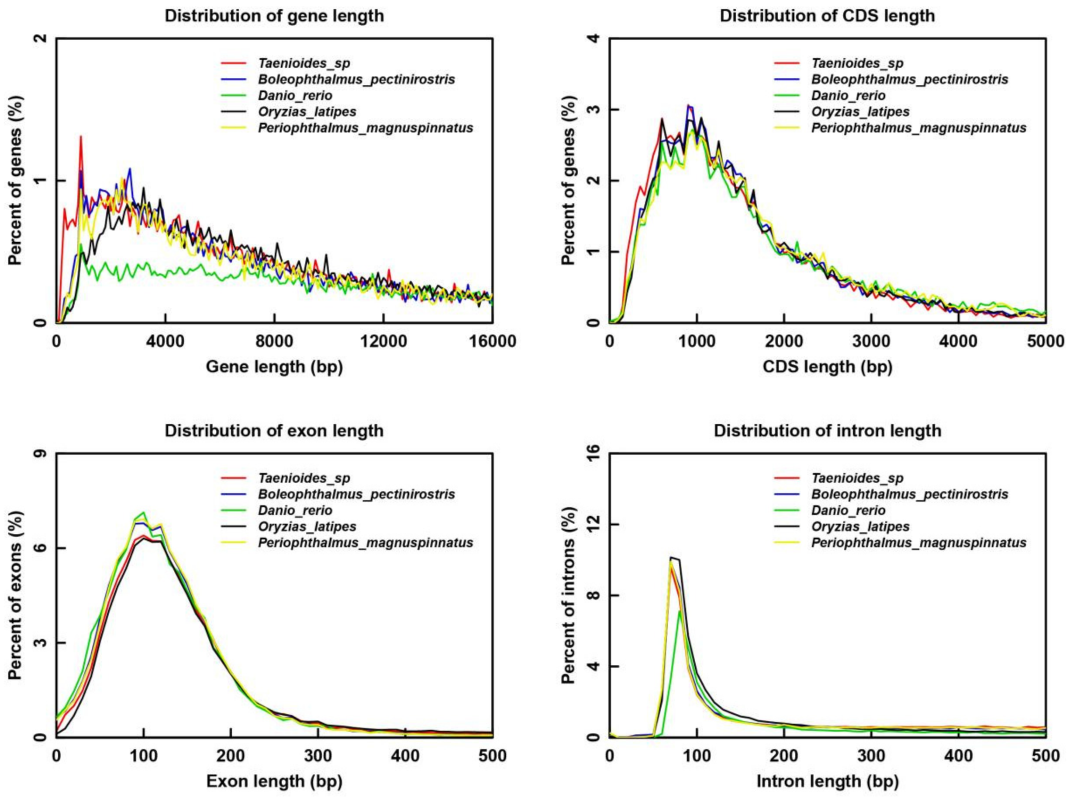

Figure S5 Annotation quality comparison of protein-coding genes.

Note: The gene length, CDS length, exon length, and intron length were compared among *Taenioides* sp and other four teleost species

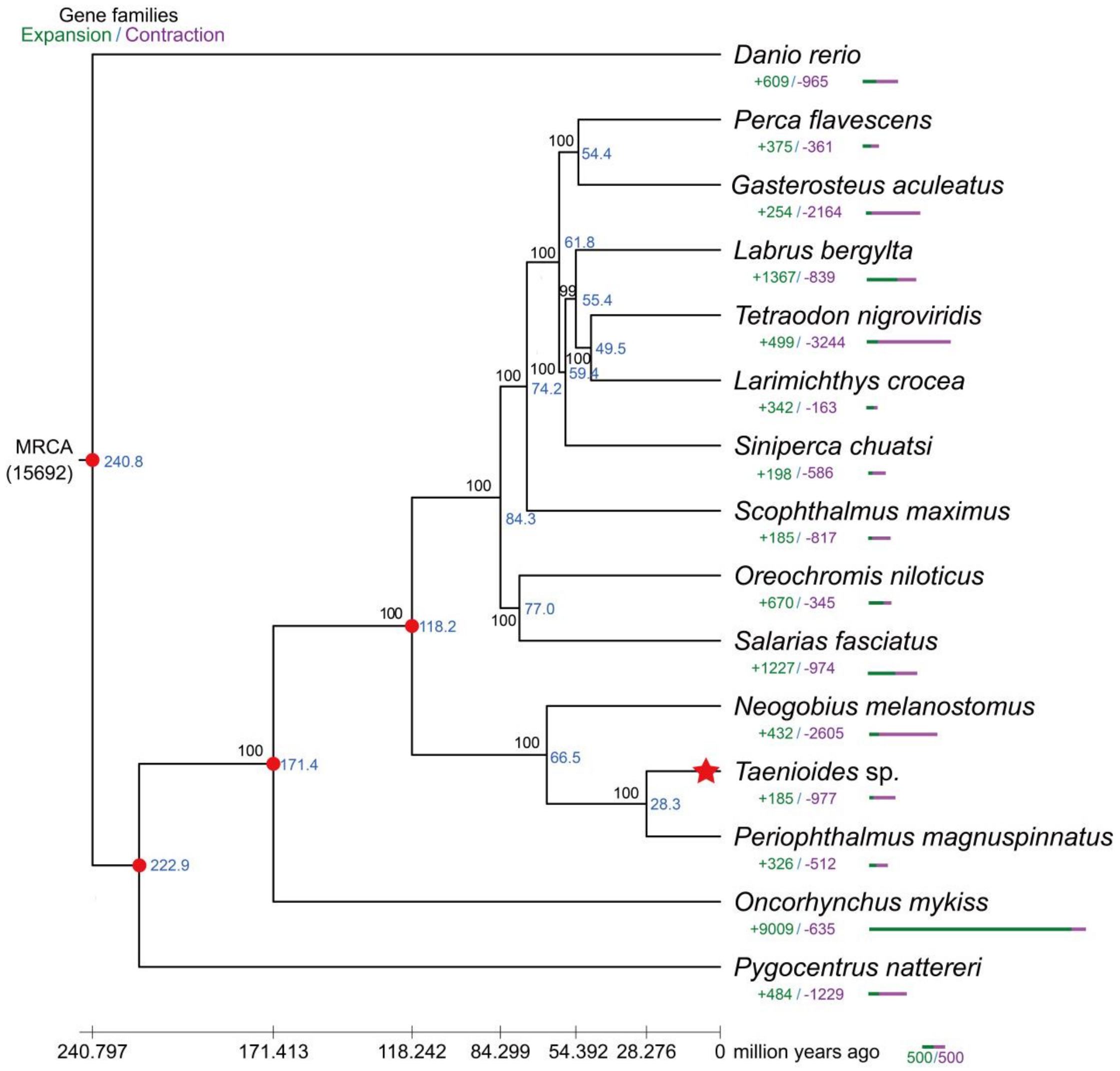

Figure S6 Phylogenetic relationship and divergence time among *Taenioides* sp and other 14 out group species, corresponding to Figure 2A in the main text.

Note: The blue number in each node represents the divergence time among species and the red circle indicates the fossil record used for calibration in the node. The black numbers in each node represent the bootstrap support for ML phylogenetic analyses. The green and purple numbers under each species name represent the expanded and contracted gene families in this node, respectively.
