# Supplementary material for: Genome Sequencing Provides Novel Insights into Mudflat Burrowing Adaptations in Eel Goby Taenioides sp. (Teleost: Amblyopinae)
Source: Int J Mol Sci. 2023 Aug 17;24(16):12892. doi: 10.3390/ijms241612892 (PMC10454203; doi:10.3390/ijms241612892)
Supplement: Supplementary file 1 [file ijms-24-12892-s001.zip › ijms-2550818-Supplementary Tables.pdf]

Table S1 Statistics of short read dataproduced by MGIDNB T7 platform

Read number	Base count (Gb)	Mean length (bp)	Q20 (%)	Q30 (%)	GC content (%)
521,365,080	78.20	150	97.25	90.68	40.87

Table S2 Statistics of the genome survey for *Taenioides* sp based on 17-mer analysis

K-mer number	K-mer depth	Genome size (Mb)	Revised genome size (Mb)	Heterozygous ratio (%)	Repeat (%)
67,447,918,210	88	766	755	0.60	45.32

Table S3 Statistics of the HiFilong readsdata produced by the PacBioSequel IIplatform

Read number	Base count (Gb)	Mean length (bp)	N50 (bp)	N90 (bp)	GC Content (%)
2,019,045	37,047,609,214	18,349.17	18,569	13,738	39.89

Table S4 Statistics for Hi-C reads data produced MGIDNB T7 platform

Read number	Base count (Gb)	Mean length (bp)	Q20 (%)	Q30 (%)	GC Content (%)
787,856,392	118.16	150	98.86	95.84	39.89

Table S5 Statistics of the pseudochromosome assemblies for *Taenioides* sp based on Hi-C data

Chromosome	Chr length (bp)	Length(bp)/Percentage(%)	Contig number	Gene number
chr1	43,849,507	5.67055	16	1,405
chr2	36,838,452	4.76389	16	1,136
chr3	37,267,491	4.81937	11	1,127
chr4	35,891,044	4.64137	3	1,144
chr5	35,514,071	4.59262	6	1,102
chr6	37,216,381	4.81276	11	1,195
chr7	38,191,833	4.93891	15	983
chr8	36,467,273	4.71589	5	1,052
chr9	33,492,621	4.33121	13	978
chr10	34,184,133	4.42064	7	876
chr11	34,473,377	4.45804	6	1,005
chr12	32,631,565	4.21986	14	1,086
chr13	31,731,700	4.10349	6	911
chr14	32,878,221	4.25176	7	1,007
chr15	33,700,152	4.35805	4	895
chr16	34,207,569	4.42367	17	916
chr17	30,406,762	3.93216	12	1,094
chr18	29,825,879	3.85704	6	929
chr19	27,503,843	3.55675	11	788
chr20	27,897,921	3.60772	2	897
chr21	27,022,322	3.49448	8	642
chr22	26,366,292	3.40965	6	736
chr23	18,787,286	2.42954	2	456

Table S6 The mapping ratio of the short reads to the assembled genome of *Taenioides* sp

Mapping rate (%)	Paired mapping rate (%)	Average sequencing depth	Coverage (%)	Coverage at least 4X (%)	Coverage at least 10X (%)	Coverage at least 20X (%)
99.64	92.23	95.11	99.80	99.43	98.83	97.79

Table S7 The mapping ratio of the PacBioHiFi long reads tothe assembled genome of *Taenioides* sp

Mapping rate (%)	Average sequencing depth	Coverage (%)	Coverage at least 4X (%)	Coverage at least 10X (%)	Coverage at least 20X (%)
99.97	47.56	100.00	99.83	99.25	97.12

Table S8. Statistics for genome synteny among *Taenioides* sp and three other telost species

Species pairs (A/B)	Align length (A/B)	Genome size (A)	The ratio in A (%)	Genome size (B)	The ratio in B (%)
Taenioides_sp/ *Danio_rerio*	1,353,139	773,284,894	0.17	1,679,203,469	0.08
Taenioides_sp / *Larimichthys_crocea*	360,528	773,284,894	0.05	657,939,657	0.05
Taenioides_sp/ *Salarias_fasciatus*	687,455	773,284,894	0.09	797,507,141	0.09

Table S9.Statistics of the annotated repeat sequences in the assembled genome of *Taenioides* sp

Type	Repeat Size (bp)	% of genome
Trf	62,097,661	8.03
Repeatmasker	94,388,556	12.21
Proteinmask	42,770,606	5.53
De novo	288,666,776	37.33
Total	365,871,704	47.31

Table S10 Statistics of the annotated TEs in the assembled genome of *Taenioides* sp

Terms	RepBase TEs		TE Proteins		De novo		Combined TEs	
	Length (bp)	% in Genome	Length (bp)	% in Genome	Length (bp)	% in Genome	Length (bp)	% in Genome
DNA	38,687,948	5.00	8,865,091	1.15	76,930,338	9.95	96,964,695	12.54
LINE	42,616,593	5.51	28,877,153	3.73	59,418,947	7.68	79,156,087	10.24
SINE	4,496,652	0.58	0	0	4,682,110	0.61	7,851,677	1.02
LTR	10,862,485	1.40	5,043,643	0.65	37,990,036	4.91	46,801,379	6.05
Satellite	3,591,066	0.46	0	0.00	1,365,041	0.18	4,885,960	0.63
Simplerepeat	0	0.00	0	0.00	575,011	0.07	575,011	0.07
Other	33,469	0.00	0	0.00	0	0.00	33,469	0.00
Unknown	918,627	0.12	4,545	0.00	121,960,265	15.77	122,756,741	15.87
Total	94,388,556	12.21	42,770,606	5.53	288,666,776	37.33	334,958,026	43.32

Table S11. Statistics of the TEs content in the assembled genome of *Taenioides* sp

Species		*Taenioides* sp.		
Types	Types	length	ratio in TEs	ratio in genome
DNA	Academ	1,813,369	0.541372011	0.234502059
DNA	CMC	12,053,059	3.59837892	1.558682847
DNA	CMC-Chapaev	191,981	0.057314942	0.024826684
DNA	Crypton	1,673,431	0.499594239	0.216405495
DNA	DNA	2,867,084	0.855953217	0.370766844
DNA	Dada	327,791	0.097860321	0.042389422
DNA	Ginger	1,015,780	0.303255907	0.13135909
DNA	Helitron	3,427,403	1.02323358	0.443226426
DNA	IS3EU	476,915	0.142380526	0.061673906
DNA	Kolobok	594,240	0.177407303	0.076846193
DNA	MULE	1,277,353	0.381347184	0.165185304
DNA	Maverick	1,447,145	0.432037714	0.187142541
DNA	Merlin	254,947	0.076113119	0.03296935
DNA	Novosib	897,264	0.267873563	0.116032785
DNA	P	365,467	0.109108298	0.047261624
DNA	PIF	6,296,280	1.879722088	0.814225139
DNA	PiggyBac	433,449	0.129403975	0.056052951
DNA	Sola	1,600,626	0.47785868	0.206990465
DNA	TcMar	20,818,795	6.215344426	2.692254195
DNA	Zator	2,021	0.000603359	0.000261353
DNA	Zisupton	850,694	0.253970329	0.110010425
DNA	hAT	45,016,993	13.43959228	5.821527531
LINE	CR1	783,206	0.233822133	0.101282982
LINE	CRE	85,180	0.025430052	0.011015345
LINE	Deceiver	892	0.000266302	0.000115352
LINE	Dong-R4	2,668,860	0.796774459	0.345132825
LINE	Dualen	1,697	0.000506631	0.000219453
LINE	Genie	138	4.11992E-05	1.78459E-05
LINE	I	359,683	0.107381514	0.046513646
LINE	L1	5,763,413	1.720637379	0.745315607
LINE	L2	22,067,031	6.58799888	2.853674134
LINE	LINE	36,357	0.010854196	0.004701631
LINE	Penelope	13,632,157	4.069810526	1.762889345
LINE	Proto1	27,150	0.008105493	0.003510996
LINE	Proto2	144,691	0.043196756	0.018711215
LINE	R1	507,683	0.151566155	0.065652776
LINE	R2	373,496	0.111505314	0.048299922
LINE	RTE	13,938,293	4.161205858	1.802478376
LINE	Rex-Babar	21,325,198	6.366528444	2.757741444
LINE	Tad1	14,638	0.0043701	0.001892963

LTR	Caulimoviru	1,170	0.000349297	0.000151303
LTR	Caulimovirus	24,348	0.00726897	0.003148645
LTR	Copia	570,343	0.170272976	0.07375587
LTR	DIRS	789,108	0.235584145	0.102046219
LTR	ERV	253,073	0.075553646	0.032727007
LTR	ERV1	3,247,760	0.969602084	0.419995273
LTR	ERV4	66,226	0.019771432	0.008564243
LTR	ERVK	436,055	0.130181983	0.056389955
LTR	ERVL	125,611	0.03750052	0.016243819
LTR	Gypsy	17,113,325	5.109095371	2.213068577
LTR	LTR	207,226	0.061866259	0.026798144
LTR	Ngaro	21,763,663	6.49742992	2.814443056
LTR	Pao	2,801,448	0.836357926	0.362278899
LTR	Viper	1,633	0.000487524	0.000211177
LTR	Copia	104	3.10487E-05	1.34491E-05
SINE	5S	287,623	0.085868371	0.037194959
SINE	5S-Core	8,732	0.002606894	0.001129209
SINE	5S-Deu	211,027	0.063001028	0.027289683
SINE	5S-Sauria	157,528	0.047029176	0.020371276
SINE	7SL	141	4.20948E-05	1.82339E-05
SINE	Alu	2,877	0.000858914	0.000372049
SINE	B2	585	0.000174649	7.56513E-05
SINE	B4	2,717	0.000811146	0.000351358
SINE	Ceph	492	0.000146884	6.36247E-05
SINE	ID	1,448	0.000432293	0.000187253
SINE	MIR	4,716,608	1.408119118	0.609944412
SINE	SINE	1,375,157	0.410546066	0.177833165
SINE	U	4,081	0.001218362	0.000527749
SINE	tRNA	1,179,418	0.352109192	0.152520502
SINE	tRNA-Ceph	73	2.17938E-05	9.44025E-06
SINE	tRNA-Core	2,947	0.000879812	0.000381101
SINE	tRNA-Deu	19,201	0.00573236	0.002483043
SINE	tRNA-Sauria	3,405	0.001016545	0.000440329
SINE	tRNA-V	268,031	0.08001928	0.034661352
SINE	tRNA-V-Core	33,594	0.010029316	0.004344324
Satellite	Satellite	4,868,557	1.453482712	0.62959422
Unknown	Helitro	4,545	0.001356886	0.000587752
Unknown	Unknown	122,751,321	36.64677705	15.87400995
Unknown	centromeric	875	0.000261227	0.000113154

Table S12. Statistics of annotated protein-coding genes in *Taenioides* sp

Total number of genes	Average length of gene (bp)	Average length of CDS (bp)	Average number of exon	Average length of exon (bp)	Average length of intron (bp)	Average length of intergenic region (bp)	Rate of single exon gene(%)
22,399	14,624.84	1,686.47	10.15	240.91	1,330.86	20,087.17	8.23

Table S13 GO enrichment of gene families that were expanded in *Taenioides* sp (P<0.05)

GO ID	Description	Count	GeneRatio	BgRatio	P value	P.adjust
GO:0071526	semaphorin-plexin signaling pathway	16	16/138	19/15263	7.71E-31	3.32E-29
GO:0017154	semaphorin receptor activity	16	16/138	21/15263	1.59E-29	3.43E-28
GO:0006352	DNA-templated transcription, initiation	9	9/138	25/15263	5.61E-13	8.04E-12
GO:0051262	protein tetramerization	7	7/138	15/15263	2.57E-11	2.76E-10
GO:0015074	DNA integration	9	9/138	39/15263	5.23E-11	4.49E-10
GO:0005085	guanyl-nucleotide exchange factor activity	9	9/138	62/15263	4.20E-09	3.01E-08
GO:0000786	nucleosome	9	9/138	76/15263	2.65E-08	1.63E-07
GO:0007264	small GTPase mediated signal transduction	9	9/138	132/15263	3.08E-06	1.65E-05
GO:0005261	cation channel activity	4	4/138	13/15263	4.29E-06	2.05E-05
GO:0030414	peptidase inhibitor activity	4	4/138	14/15263	5.97E-06	2.57E-05
GO:0005525	GTP binding	14	14/138	433/15263	3.80E-05	1.48E-04
GO:0007286	spermatid development	3	3/138	10/15263	8.28E-05	2.46E-04
GO:0046718	viral entry into host cell	3	3/138	10/15263	8.28E-05	2.46E-04
GO:0050839	cell adhesion molecule binding	3	3/138	10/15263	8.28E-05	2.46E-04
GO:0016757	transferase activity, transferring glycosyl groups	5	5/138	50/15263	8.59E-05	2.46E-04
GO:0006879	cellular iron ion homeostasis	3	3/138	14/15263	2.44E-04	6.58E-04
GO:0005911	cell-cell junction	3	3/138	16/15263	3.72E-04	8.41E-04
GO:0042803	protein homodimerization activity	3	3/138	16/15263	3.72E-04	8.41E-04
GO:0046982	protein heterodimerization activity	3	3/138	16/15263	3.72E-04	8.41E-04
GO:0016746	transferase activity, transferring acyl groups	4	4/138	40/15263	4.54E-04	9.76E-04
GO:0008152	metabolic process	8	8/138	236/15263	1.37E-03	2.81E-03
GO:0008237	metallopeptidase activity	4	4/138	71/15263	3.89E-03	7.61E-03
GO:0034220	ion transmembrane transport	4	4/138	83/15263	6.77E-03	1.27E-02
GO:0006812	cation transport	3	3/138	45/15263	7.78E-03	1.39E-02
GO:0008009	chemokine activity	2	2/138	23/15263	1.81E-02	3.12E-02
GO:0004222	metalloendopeptidase activity	4	4/138	121/15263	2.41E-02	3.99E-02
GO:0005615	extracellular space	3	3/138	73/15263	2.84E-02	4.53E-02
GO:0007155	cell adhesion	4	4/138	134/15263	3.33E-02	5.13E-02
GO:0004867	serine-type endopeptidase inhibitor activity	2	2/138	38/15263	4.62E-02	6.84E-02

Table S14 Enrichment of KEGG pathways of gene families that were expanded in *Taenioides* sp (P<0.05)

KEGG ID	Description	Count	GeneRatio	BgRatio	P value	P.adjust
ko04640	Hematopoietic cell lineage	14	14/99	96/8417	4.10E-12	3.98E-10
ko04662	B cell receptor signaling pathway	14	14/99	112/8417	3.53E-11	1.71E-09
ko05330	Allograft rejection	11	11/99	65/8417	1.79E-10	5.79E-09
ko05320	Autoimmune thyroid disease	11	11/99	67/8417	2.52E-10	6.12E-09
ko04145	Phagosome	16	16/99	205/8417	1.63E-09	3.16E-08
ko05152	Tuberculosis	16	16/99	222/8417	5.20E-09	8.40E-08
ko04360	Axon guidance	17	17/99	275/8417	1.67E-08	2.31E-07
ko05416	Viral myocarditis	11	11/99	107/8417	4.13E-08	5.01E-07
ko04514	Cell adhesion molecules	15	15/99	242/8417	1.23E-07	1.33E-06
ko04072	Phospholipase D signaling pathway	14	14/99	223/8417	2.89E-07	2.80E-06
ko05150	Staphylococcus aureus infection	8	8/99	68/8417	1.14E-06	1.00E-05
ko05310	Asthma	5	5/99	21/8417	3.56E-06	2.88E-05
ko04610	Complement and coagulation cascades	8	8/99	91/8417	1.05E-05	7.82E-05
ko05332	Graft-versus-host disease	6	6/99	52/8417	2.99E-05	2.07 E-04
ko04650	Natural killer cell mediated cytotoxicity	8	8/99	118/8417	6.95E-05	4.48 E-04
ko05322	Systemic lupus erythematosus	8	8/99	119/8417	7.38E-05	4.48 E-04
ko04672	Intestinal immune network for IgA production	5	5/99	39/8417	8.53E-05	4.87 E-04
ko05340	Primary immunodeficiency	5	5/99	40/8417	9.66E-05	5.20 E-04
ko04940	Type I diabetes mellitus	6	6/99	66/8417	1.17E-04	5.96 E-04
ko05143	African trypanosomiasis	5	5/99	48/8417	2.33E-04	1.09 E-03
ko05146	Amoebiasis	8	8/99	141/8417	2.41E-04	1.09 E-03
ko04720	Long-term potentiation	7	7/99	107/8417	2.51E-04	1.09 E-03
ko00512	Mucin type O-glycan biosynthesis	5	5/99	49/8417	2.57E-04	1.09 E-03
ko05204	Chemical carcinogenesis	5	5/99	50/8417	2.83E-04	1.15 E-03
ko04670	Leukocyte transendothelial migration	9	9/99	197/8417	4.93E-04	1.87 E-03
ko05323	Rheumatoid arthritis	6	6/99	86/8417	5.00E-04	1.87 E-03
ko00514	Other types of O-glycan biosynthesis	5	5/99	59/8417	6.16E-04	2.21 E-03
ko04978	Mineral absorption	5	5/99	71/8417	1.43E-03	4.97 E-03
ko04261	Adrenergic signaling in cardiomyocytes	9	9/99	230/8417	1.49E-03	4.97 E-03
ko04726	Serotonergic synapse	7	7/99	156/8417	2.35E-03	7.60 E-03
ko04664	Fc epsilon RI signaling pathway	5	5/99	83/8417	2.86E-03	8.95 E-03
ko00983	Drug metabolism - other enzymes	5	5/99	86/8417	3.34E-03	1.01 E-02

ko05140	Leishmaniasis	5	5/99	87/8417	3.51E-03	1.03 E-02
ko00071	Fatty acid degradation	4	4/99	55/8417	3.88E-03	1.11 E-02
ko04015	Rap1 signaling pathway	10	10/99	324/8417	4.64E-03	1.29 E-02
ko05221	Acute myeloid leukemia	5	5/99	110/8417	9.41E-03	2.48 E-02
ko05169	Epstein-Barr virus infection	8	8/99	256/8417	1.04E-02	2.62 E-02
ko01212	Fatty acid metabolism	4	4/99	73/8417	1.06E-02	2.62 E-03
ko04064	NF-kappa B signaling pathway	5	5/99	114/8417	1.09E-02	2.64 E-02
ko03320	PPAR signaling pathway	4	4/99	87/8417	1.91E-02	4.51 E-02
ko05170	Human immunodeficiency virus 1 infection	8	8/99	289/8417	2.02E-02	4.67 E-02
ko04024	cAMP signaling pathway	9	9/99	353/8417	2.27E-02	5.13 E-02
ko04216	Ferroptosis	3	3/99	56/8417	2.79E-02	6.15 E-02
ko04666	Fc gamma R-mediated phagocytosis	5	5/99	164/8417	4.38E-02	9.43 E-02
ko04320	Dorso-ventral axis formation	2	2/99	30/8417	4.81E-02	9.93 E-02

Table S15 GO enrichment of gene families that were contracted in *Taenioides* sp (P<0.05)

GO ID	Description	Count	GeneRatio	BgRatio	P value	P.adjust
GO:0007156	homophilic cell adhesion via plasma membrane adhesion molecules	22	22/88	106/15263	6.84E-29	1.30E-27
GO:0005622	intracellular	21	21/88	374/15263	1.90E-15	1.80E-14
GO:0005328	neurotransmitter:sodium symporter activity	4	4/88	39/15263	7.27E-05	4.61E-04
GO:0006836	neurotransmitter transport	4	4/88	44/15263	1.17E-04	5.59E-04
GO:0005615	extracellular space	4	4/88	73/15263	8.29E-04	3.15E-03
GO:0004866	endopeptidase inhibitor activity	2	2/88	13/15263	2.46E-03	7.79E-03
GO:0004553	hydrolase activity, hydrolyzing O-glycosyl compounds	2	2/88	30/15263	1.29E-02	3.49E-02
GO:0005887	integral component of plasma membrane	3	3/88	118/15263	3.07E-02	7.30E-02
GO:0005525	GTP binding	6	6/88	433/15263	3.89E-02	8.12E-02
GO:0008146	sulfotransferase activity	2	2/88	57/15263	4.27E-02	8.12E-02

Table S16 Enrichment of KEGG pathways with contracted gene families in *Taenioides* sp (P<0.05)

KEGG ID	Description	Count	GeneRatio	BgRatio	P value	P.adjust
ko04621	NOD-like receptor signaling pathway	12	12/31	168/8417	2.74E-13	1.45E-11
ko04928	Parathyroid hormone synthesis, secretion and action	11	11/31	166/8417	7.59E-12	2.01E-10
ko04610	Complement and coagulation cascades	3	3/31	91/8417	4.41E-03	5.85E-02
ko00350	Tyrosine metabolism	2	2/31	37/8417	8.07E-03	8.55E-02
ko04623	Cytosolic DNA-sensing pathway	2	2/31	44/8417	1.13E-02	8.90E-02
ko00620	Pyruvate metabolism	2	2/31	51/8417	1.50E-02	8.90E-02
ko00980	Metabolism of xenobiotics by cytochrome P450	2	2/31	53/8417	1.61E-02	8.90E-02
ko00982	Drug metabolism - cytochrome P450	2	2/31	53/8417	1.61E-02	8.90E-02
ko00071	Fatty acid degradation	2	2/31	55/8417	1.73E-02	8.90E-02
ko00830	Retinol metabolism	2	2/31	57/8417	1.85E-02	8.90E-02
ko05320	Autoimmune thyroid disease	2	2/31	67/8417	2.50E-02	1.05E-01
ko04622	RIG-I-like receptor signaling pathway	2	2/31	71/8417	2.79E-02	1.05E-01
ko04978	Mineral absorption	2	2/31	71/8417	2.79E-02	1.05E-01
ko00010	Glycolysis / Gluconeogenesis	2	2/31	77/8417	3.24E-02	1.14E-01

Table S17 Candidate positively selected genes in *Taenioides* sp (P<0.05)

Gene ID	P value	Corrected P value	Site_Num	Swissprot Annotation
Tas_10G0000950.1	9.91E-07	2.16E-05	3	*WDR11*
Tas_10G0001140.1	3.31E-07	7.48E-06	15	*MCMBP*
Tas_10G0002210.1	4.31 E-03	4.47E-02	2	*ARFGEF3*
Tas_10G0002460.1	0	0.00E-00	73	*EXO1*
Tas_10G0003640.1	4.33E-08	1.14E-06	14	*SPEF1*
Tas_10G0003690.1	2.18E-10	7.29E-09	13	*ECHS1*
Tas_10G0004090.1	5.51 E-04	7.21E-03	9	*MORN4*
Tas_10G0004120.1	1.19E-10	4.13E-09	19	*HPS1*
Tas_10G0005320.1	2.99E-11	1.08E-09	8	*SUPT3H*
Tas_10G0005360.1	1.63E-07	3.99E-06	19	*MCM8*
Tas_10G0006770.1	8.47E-12	3.43E-10	16	*RAD30*
Tas_11G0000580.1	5.05E-11	1.79E-09	33	*RABGGTA*
Tas_11G0000780.1	0	0.00E+00	90	*SCFD2*
Tas_11G0002870.1	3.85E-05	6.32E-04	4	*STIL*
Tas_11G0003210.1	3.09 E-04	4.17E-03	5	*IFI30*
Tas_11G0004160.1	4.21E-10	1.33E-08	20	*NR2C2AP*
Tas_11G0006280.1	1.07 E-04	1.64E-03	22	*MINDY4*
Tas_11G0006650.1	4.23E-06	8.36E-05	3	*VTG*
Tas_11G0007390.1	0	0.00E-00	78	*GMD*
Tas_11G0007610.1	1.67 E-04	2.48E-03	3	*C8orf82*
Tas_12G0002890.1	6.74E-06	1.29E-04	13	*LRRCC*
Tas_12G0007060.1	0	0.00E-00	60	*CACYBP*
Tas_12G0007140.1	6.58E-10	1.98E-08	8	*RAB6B*
Tas_12G0008160.1	2.08E-06	4.36E-05	13	*ASB10*
Tas_12G0010440.1	6.43 E-04	8.30E-03	5	*ARTD2*
Tas_12G0010770.1	1.74 E-03	2.00E-02	8	*ZNF622*
Tas_13G0001010.1	3.40 E-03	3.65E-02	107	*FAM176B*
Tas_13G0004210.1	1.79E-09	5.27E-08	13	*MATN1*
Tas_13G0006730.1	5.24 E-04	6.91E-03	1	*HAMAP*
Tas_13G0007010.1	2.41 E-03	2.68E-02	2	*TEKT2*
Tas_13G0008470.1	1.79E-05	3.16E-04	6	*DAZ1*
Tas_13G0008610.1	4.70 E-03	4.78E-02	4	*FAM221A*
Tas_14G0000170.1	3.95E-13	1.86E-11	13	*DDX46*
Tas_14G0000670.1	2.20 E-03	2.46E-02	4	*LRWD1*
Tas_14G0001700.1	6.31E-05	1.01E-03	13	*F9*
Tas_14G0003520.1	0	0.00E-00	62	*DDX52*
Tas_14G0006060.1	2.31E-05	3.98E-04	7	*SHPK*
Tas_14G0006250.1	3.20E-08	8.58E-07	31	*SUPT4H1*
Tas_15G0001270.1	6.81E-05	1.08E-03	6	*SLC38A11*
Tas_15G0001320.1	7.39E-08	1.92E-06	12	*IFIH1*
Tas_15G0004100.1	1.57 E-03	1.84E-02	3	*TMEM30A*
Tas_15G0004350.1	2.92 E-03	3.18E-02	3	*UCHL3*

Tas_15G0004390.1	0	0.00E-00	77	*GPR18*
Tas_15G0004820.1	7.19 E-04	9.14E-03	7	*TBCCD1*
Tas_15G0005690.1	2.44E-15	1.77E-13	57	*SLC12A8*
Tas_15G0006810.1	0	0.00E-00	129	*COPS8*
Tas_15G0007130.1	2.12E-12	9.02E-11	27	*LMLN*
Tas_15G0007620.1	6.75E-06	1.29E-04	12	*HSPBAP1*
Tas_15G0008580.1	6.23E-09	1.78E-07	16	*TMEM177*
Tas_16G0000120.1	1.76 E-03	2.02E-02	0	*RPB11a*
Tas_16G0000230.1	1.01 E-04	1.56E-03	15	*PNP*
Tas_16G0000470.1	1.40E-05	2.49E-04	9	*PXK*
Tas_16G0002680.1	2.23E-07	5.18E-06	17	*HISRS*
Tas_16G0003400.1	1.73 E-03	2.00E-02	3	*MRPL11*
Tas_16G0003670.1	4.81 E-03	4.87E-02	1	*TRM112*
Tas_16G0003740.1	3.63E-12	1.50E-10	18	*TRPT1*
Tas_16G0005060.1	4.22E-05	6.86E-04	5	*NUDCD2*
Tas_16G0005820.1	3.47E-05	5.76E-04	10	*INTS10*
Tas_16G0006810.1	1.16 E-04	1.76E-03	0	*NXT2*
Tas_16G0007160.1	2.81 E-03	3.08E-02	11	*MTUS1*
Tas_16G0007750.1	2.04 E-03	2.31E-02	8	*C1QTNF1*
Tas_17G0003540.1	1.78 E-03	2.03E-02	6	*PGLS*
Tas_18G0000740.1	8.81 E-04	1.09E-02	10	*ID3A*
Tas_18G0001050.1	8.43E-06	1.53E-04	3	*BDKRB2*
Tas_18G0001150.1	1.25E-06	2.69E-05	10	*DPH6*
Tas_18G0001760.1	3.07 E-04	4.17E-03	3	*RRH*
Tas_18G0001990.1	2.62E-05	4.38E-04	5	*MSH4*
Tas_18G0002610.1	0	0.00E-00	31	*FACM*
Tas_18G0002670.1	3.00E-09	8.71E-08	18	*DLK1*
Tas_18G0004170.1	8.36E-06	1.53E-04	2	*CCDC28B*
Tas_18G0004180.1	2.36E-05	4.03E-04	4	*TMEM39B*
Tas_18G0004640.1	7.07E-06	1.34E-04	5	*KCP3*
Tas_18G0005800.1	1.29 E-03	1.53E-02	13	*TRAPPC12*
Tas_18G0006860.1	3.70 E-03	3.95E-02	5	*FBLN5*
Tas_18G0006870.1	7.84E-06	1.45E-04	4	*TRIP11*
Tas_19G0001420.1	3.38E-13	1.64E-11	20	*SNW1*
Tas_19G0002900.1	0	0.00E-00	57	*NBAS*
Tas_19G0003520.1	8.70 E-04	1.09E-02	8	*GATA4*
Tas_19G0003540.1	7.42E-06	1.39E-04	17	*FDFT1*
Tas_19G0004240.1	3.82 E-03	4.05E-02	6	*CEP57L1*
Tas_19G0004330.1	4.13 E-03	4.31E-02	4	*CCDC164*
Tas_19G0006730.1	2.19 E-03	2.46E-02	4	*MRPL19*
Tas_1G0000210.1	0	0.00E-00	33	*COG5*
Tas_1G0004660.1	2.28E-05	3.97E-04	15	*AMDHD1*
Tas_1G0006530.1	5.34E-06	1.05E-04	6	*NAIF1*
Tas_1G0007860.1	0	0.00E-00	46	*CWC27*

Tas_1G0007970.1	4.44E-16	3.51E-14	31	*PAR3*
Tas_1G0008730.1	0	0.00E-00	78	*PTCD2*
Tas_1G0009310.1	1.27 E-03	1.52E-02	7	*NUP54*
Tas_1G0009530.1	1.90 E-04	2.69E-03	5	*PRLR*
Tas_1G0009670.1	2.50E-05	4.22E-04	6	*NOP14*
Tas_1G0010280.1	5.47E-14	3.07E-12	19	*OAZ2*
Tas_1G0011230.1	2.64 E-03	2.90E-02	2	*LGP85*
Tas_1G0011770.1	0	0.00E-00	58	*NOC4*
Tas_1G0012870.1	2.61E-07	5.97E-06	6	*GTF3C3*
Tas_20G0004590.1	1.03 E-03	1.26E-02	6	*CCDC186*
Tas_20G0006410.1	7.99E-07	1.78E-05	22	*MED11*
Tas_20G0007500.1	2.00E-06	4.25E-05	7	*PIPPIN*
Tas_20G0008100.1	5.88E-15	3.94E-13	46	*SPATA20*
Tas_20G0008320.1	6.40E-10	1.95E-08	9	*FDXR*
Tas_20G0008440.1	2.56E-14	1.49E-12	51	*SAP30BP*
Tas_21G0000470.1	2.29E-13	1.17E-11	12	*RBCK1*
Tas_21G0000840.1	1.10E-11	4.18E-10	24	*ZC2HC1A*
Tas_21G0001040.1	1.85E-14	1.11E-12	14	*FEN1*
Tas_21G0001340.1	6.79 E-04	8.69E-03	2	*RBM40*
Tas_21G0001650.1	2.22E-13	1.17E-11	17	*MBTPS2*
Tas_21G0003510.1	3.85E-10	1.24E-08	9	*TMPRSS7*
Tas_21G0003990.1	0	0.00E-00	44	*MyD88*
Tas_21G0004100.1	4.59 E-03	4.70E-02	1	*CPB*
Tas_21G0004140.1	8.84E-12	3.50E-10	11	*PLSCR1*
Tas_21G0005970.1	3.87 E-03	4.08E-02	4	*IFT74*
Tas_22G0001660.1	8.42E-07	1.86E-05	7	*FAM28A*
Tas_22G0002760.1	5.83E-13	2.67E-11	26	*SEPSECS*
Tas_22G0007140.1	2.13E-04	2.97E-03	2	*TCP1*
Tas_23G0002710.1	8.56E-08	2.19E-06	57	*HDHD1*
Tas_2G0002120.1	2.20E-07	5.17E-06	13	*LETMD1*
Tas_2G0003170.1	1.10E-11	4.18E-10	76	*AURKAIP1*
Tas_2G0003880.1	3.08 E-03	3.33E-02	3	*FBXW12*
Tas_2G0004190.1	1.83E-14	1.11E-12	24	*EXOSC10*
Tas_2G0006220.1	0	0.00E-00	41	*B4GALT3*
Tas_2G0006340.1	1.61E-14	1.04E-12	18	*UVPP*
Tas_2G0007020.1	7.14E-05	1.12E-03	13	*EIF2D*
Tas_2G0009040.1	3.48E-06	7.05E-05	9	*XPCC*
Tas_2G0009520.1	1.92 E-04	2.70E-03	21	*CELA2A*
Tas_3G0000150.1	1.72 E-04	2.49E-03	9	*PHF13*
Tas_3G0003390.1	1.72E-11	6.36E-10	45	*ABHD14B*
Tas_3G0004460.1	2.22E-16	1.84E-14	34	*RPUSD4*
Tas_3G0005990.1	8.38 E-04	1.06E-02	3	*RFESD*
Tas_3G0007940.1	4.19E-06	8.36E-05	8	*BLOC1S1*
Tas_3G0007950.1	1.04E-12	4.52E-11	42	*PXMP4*

Tas_3G0008750.1	2.95E-10	9.70E-09	14	*CIDEC*
Tas_3G0008780.1	2.61 E-04	3.61E-03	5	*HMCES*
Tas_4G0003020.1	4.48 E-03	4.61E-02	3	*CSF3R*
Tas_4G0005330.1	4.16 E-04	5.53E-03	6	*NSMCE2*
Tas_4G0005430.1	1.08E-07	2.71E-06	4	*CMC1*
Tas_4G0006350.1	1.44E-10	4.91E-09	15	*SMG9*
Tas_4G0006870.1	2.78E-15	1.93E-13	22	*CREB5*
Tas_4G0007120.1	0	0.00E-00	80	*BRD9*
Tas_4G0009280.1	0	0.00E-00	23	*KIF21A*
Tas_5G0000020.1	6.32E-10	1.95E-08	16	*RIOK1*
Tas_5G0002900.1	2.82E-06	5.77E-05	15	*IDUA*
Tas_5G0003560.1	1.09E-07	2.71E-06	18	*POLD2*
Tas_5G0003600.1	1.80 E-04	2.58E-03	5	*C5orf34*
Tas_5G0005650.1	1.12 E-03	1.35E-02	3	*POC5*
Tas_5G0006370.1	1.11E-16	9.66E-15	18	*CDC45*
Tas_5G0006620.1	1.79E-07	4.28E-06	12	*UTP15*
Tas_5G0009120.1	2.56E-08	7.08E-07	28	*QNG1*
Tas_5G0010860.1	1.70 E-04	2.49E-03	9	*TOP3B1*
Tas_6G0000560.1	1.90 E-04	2.69E-03	3	*RRP36*
Tas_6G0002250.1	1.27 E-03	1.52E-02	5	*ANKRD49*
Tas_7G0000240.1	1.34 E-03	1.58E-02	17	*WDHD1*
Tas_7G0004770.1	0	0.00E-00	138	*DNA2*
Tas_7G0005010.1	2.86E-08	7.79E-07	8	*CSTF3*
Tas_8G0000190.1	1.02 E-03	1.25E-02	14	*FAM96A*
Tas_8G0000790.1	1.77E-07	4.28E-06	9	*DEAF1*
Tas_8G0001540.1	3.00 E-04	4.12E-03	6	*RASSF9*
Tas_8G0003360.1	1.08E-05	1.95E-04	12	*RAD52*
Tas_8G0004450.1	4.94E-05	7.96E-04	10	*MRPS35*
Tas_8G0006300.1	8.83 E-04	1.09E-02	5	*RIC8A*
Tas_8G0007450.1	3.31 E-04	4.44E-03	7	*BBS2*
Tas_8G0007720.1	6.38 E-04	8.29E-03	7	*PMLP*
Tas_8G0009260.1	1.70 E-04	2.49E-03	7	*TSPAN33*
Tas_8G0009760.1	2.20E-06	4.56E-05	9	*NEIL1*
Tas_8G0009940.1	4.13 E-03	4.31E-02	6	*SSPN*
Tas_9G0001760.1	6.09E-13	2.72E-11	45	*LHFP16*
Tas_9G0005370.1	1.44E-15	1.09E-13	64	*KIAA0753*
Tas_9G0005950.1	1.48 E-04	2.22E-03	6	*IASPP*
Tas_9G0006270.1	9.69E-05	1.51E-03	11	*GAB2*
Tas_9G0006500.1	1.93E-13	1.05E-11	19	*SMTL*

Table S18 GO enrichment of positively selected genes in *Taenioides* sp (P<0.05)

GO ID	Description	Count	Gene Ratio	BgRatio	P value	P.adjust
GO:0004518	nuclease activity	3	3/119	15/15263	1.96 E-04	1.15E-02
GO:0003684	damaged DNA binding	3	3/119	16/15263	2.40 E-04	1.15 E-02
GO:0006270	DNA replication initiation	2	2/119	11/15263	3.17 E-02	9.24 E-02
GO:0006281	DNA repair	4	4/119	82/15263	3.85 E-03	9.24 E-02
GO:0007596	blood coagulation	2	2/119	16/15263	6.73 E-03	1.17 E-01
GO:0006915	apoptotic process	3	3/119	51/15263	7.33 E-03	1.17 E-01
GO:0005840	ribosome	4	4/119	113/15263	1.18 E-02	1.37 E-01
GO:0005975	carbohydrate metabolic process	4	4/119	117/15263	1.33 E-02	1.37 E-01
GO:0006139	nucleobase-containing compound metabolic process	2	2/119	23/15263	1.37 E-02	1.37 E-01
GO:0006412	translation	4	4/119	121/15263	1.48 E-02	1.37 E-01
GO:0003735	structural constituent of ribosome	4	4/119	123/15263	1.57 E-02	1.37 E-01

Table S19 Enrichment of KEGG pathways of positively selected genes in *Taenioides* sp (P<0.05)

KEGG ID	Description	Count	GeneRatio	BgRatio	P value	P.adjust
ko03410	Base excision repair	4	4/58	33/8417	7.16E-05	1.01 E-02
ko03030	DNA replication	3	3/58	36/8417	1.89 E-03	9.60 E-02
ko03440	Homologous recombination	3	3/58	37/8417	2.04 E-03	9.60 E-02
ko04610	Complement and coagulation cascades	4	4/58	91/8417	3.48 E-03	1.23 E-01
ko03460	Fanconi anemia pathway	3	3/58	55/8417	6.32 E-03	1.78 E-01
ko03430	Mismatch repair	2	2/58	23/8417	1.08 E-02	2.53 E-01
ko03008	Ribosome biogenesis in eukaryotes	3	3/58	73/8417	1.37 E-02	2.77 E-01
ko00970	Aminoacyl-tRNA biosynthesis	2	2/58	43/8417	3.52 E-02	5.51 E-01
ko03420	Nucleotide excision repair	2	2/58	43/8417	3.52 E-02	5.51 E-01
